# Harnessing marine plant extracts for sustainable agriculture, livestock management, industrial use, and biomedical applications

**DOI:** 10.3389/fpls.2025.1686511

**Published:** 2025-11-06

**Authors:** Metin Yildirim, Madina Amangeldinova, Samet Poyraz, Mehmet Erşatır, Aizhan Mussayeva, Nataliya Kudrina

**Affiliations:** 1 Department of Biochemistry, Faculty of Pharmacy, Harran University, Sanliurfa, Türkiye; 2 A Faculty of Biology and Biotechnology, Al-Farabi Kazakh National University, Almaty, Kazakhstan; 3 Institute of Genetics and Physiology, Almaty, Kazakhstan; 4 Department of Basic Pharmaceutical Sciences, Faculty of Pharmacy, Çukurova University, Adana, Türkiye; 5 Department of Chemistry, Faculty of Art and Science, Cukurova University, Adana, Türkiye

**Keywords:** marine plant extracts, sustainable agriculture, functional feed additives, industrial applications, green technology, cancer therapy

## Abstract

Marine plants are emerging as versatile resources for bioactive compounds with antioxidant, antibacterial, anti-inflammatory, and anticancer properties. Beyond their therapeutic potential, marine extracts offer agronomic and industrial value as biostimulants, natural pigments, biodegradable packaging materials, and green corrosion inhibitors, and serve as reducing agents in the synthesis of biomedical nanoparticles. This review integrates evidence from 256 studies (2011–2025), revealing rapid growth in the field over the past two years. The findings highlight the capacity of marine extracts to enhance crop and livestock productivity, yield stable natural dyes, create smart polysaccharide-based films, and protect metals via phenolic and sulfated polysaccharide fractions. Nanoparticles synthesized from these extracts exhibit improved biological performance. By linking agricultural, industrial, and biomedical perspectives, this work underscores the multifaceted potential of marine plant extracts and outlines future priorities in molecular characterization, strain development, and scalable green processing.

## 1 Introduction

In recent years, growing concerns over the side effects of conventional drugs and the emergence of drug resistance have driven increased interest in plant-derived extracts. While terrestrial plants have long been explored, marine plants harbor a wealth of bioactive compounds that exhibit broad-spectrum activities antioxidant (Do et al., 2025), antibacterial (Ouaddi et al., 2025), anti-inflammatory and anticancer (Do et al., 2025) documented extensively in the literature. Beyond their medicinal potential, these extracts have proven useful in promoting plant and animal growth and enabling diverse industrial applications. Although several reviews address marine-derived extracts generally, work that focuses exclusively on marine plant extracts and consolidates their wide-ranging uses remains scarce. Unlike previous compilations, the present study systematically surveys marine plant extracts across agricultural and animal-agriculture contexts, as natural coloring agents, in food packaging, and for corrosion inhibition in industrial applications. It further examines nanoparticles formulated with these extracts, together with their anticancer, anti-inflammatory, antioxidant and antibacterial applications. Our literature search covers 2011–2025 and retrieved 256 studies in 2011, 13 in 2020, 26 in 2021, 22 in 2022, 22 in 2023, 81 in 2024, and 71 in 2025-placing particular emphasis on the dramatic rise in research over the last two years (**Table 1**).

**Table 1:** Number of references by publication year (2010–2025).

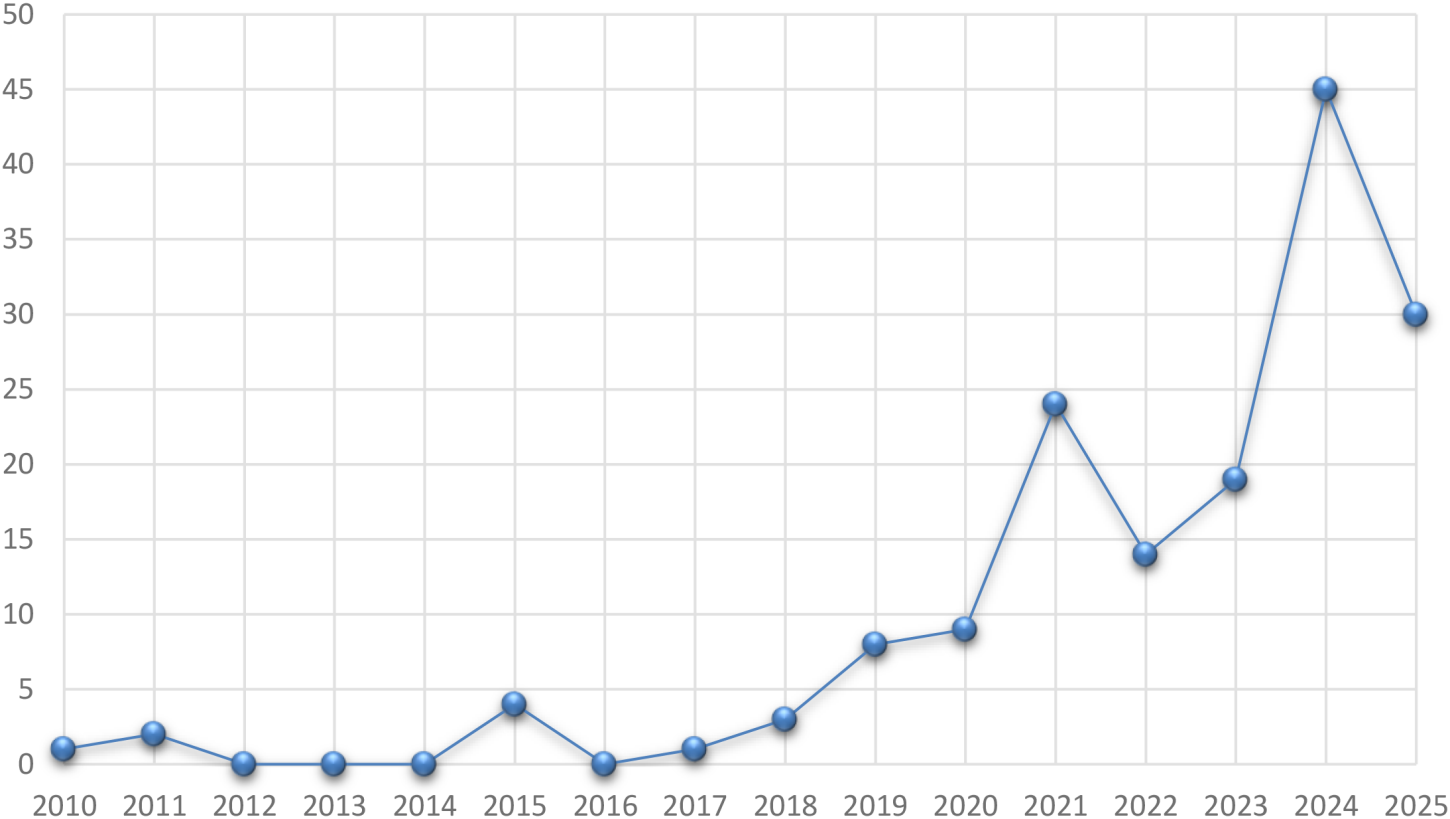 .

## 2 Agricultural applications

### 2.1 Biostimulants and biofertilizer

Agriculture plays a central role in the economy, environmental sustainability, and food security (Qian et al., 2023), as it not only provides the entire population with food products but also serves as a vital source of raw materials for the textile, pharmaceutical, and other industrial sectors. Contemporary challenges such as global warming (Timmis and Ramos, 2021), population growth, and the depletion of humus necessitate the implementation of innovative and sustainable farming practices to ensure that future generations are supplied with essential resources and a stable ecosystem (Alengebawy et al., 2021; Hou et al., 2020).

Soil degradation is one of the most pressing issues in agriculture, as soil supports freshwater systems and provides essential resources, including food, water, and raw materials (J. Wang et al., 2023). According to the latest UNCCD data (2023), land degradation affects 1.2 billion people and spans an area of 1.5 billion hectares, with this figure increasing by 100 million hectares annually (Mukherji et al., 2024). Terrestrial ecosystems have historically acted as a major sink for carbon dioxide, capturing up to one-third of anthropogenic emissions despite their doubling in recent decades, however, over the past decade, the ability of forests and soils to absorb excess CO_2_ has declined by 20%, which is attributed to intensified deforestation and shifts in the global climate system (Psistaki et al., 2024). Analytical reports identify unsustainable agricultural practices as the primary driver of forest ecosystem degradation, accounting for 80% of global forest cover loss (Singh et al., 2024).

In this context, there is growing interest in the use of promising types of biostimulants and biofertilizers derived from natural products that contain bioactive compounds and enhance the physiological processes of plants (Shahrajabian and Sun, 2022). Key nutrients include polysaccharides, organic acids, and proteins, which are often imbalanced, and the timely application of biostimulants helps support the formation of a healthy aerial biomass. Among the most promising and environmentally safe sources of such bioactive compounds are marine and freshwater algae (Michalak and Chojnacka, 2015). Due to their high content of natural polysaccharides such as laminarin, alginates, and fucoidans, along with amino acids, vitamins, phytohormones, and micronutrients, algae are effectively utilized in the composition of modern biostimulants and biofertilizers (Ameen et al., 2021; Menaa et al., 2021; Ślusarczyk et al., 2021). These compounds not only stimulate plant growth and germination but also enhance the resilience of agricultural crops to abiotic stresses, including drought, salinity, and temperature fluctuations. Algae-based biological products are increasingly regarded as a key component of sustainable agriculture, contributing to the restoration of soil fertility and reducing dependency on synthetic agrochemicals (Ghosh et al., 2022).

In the study, Baroud et al. (2024) demonstrated that aqueous extracts (0.5–2%) and organic amendments from brown algae *Fucus* sp*iralis*, *Bifurcaria bifurcata*, and *Cystoseira gibraltarica* significantly improved growth, yield, and fruit quality of greenhouse-grown pepper (*Capsicum annuum*). The 1% F. spiralis extract produced the longest shoots (75.8 cm), roots (33.2 cm), and highest dry biomass (9.88 g), while 0.5% *B. bifurcata* maximized flower production and 1% *C. gibraltarica* increased average fruit mass. Extracts also enhanced soluble sugar content, Brix value, and leaf mineral composition, highlighting brown algae as promising biostimulants for sustainable agriculture (Baroud et al., 2024).

In parallel, El Semary et al. (2023) demonstrated that mixotrophic cultivation of *Phormidium* sp. supplemented with low-cost carbon sources such as sugarcane molasses and *Lepidium sativum* extract led to a significant increase in biomass, chlorophyll content (up to 1.72 μg/g), soluble sugars (up to 17.5 mg/g), and proteins (up to 130.09 mg/g). When methanolic extracts of *Phormidium* sp. were applied to melon (*Cucumis melo*) seeds, a high germination rate was observed (up to 96% at 1% extract concentration), along with statistically significant increases in shoot and root length, fresh and dry biomass, photosynthetic pigment content, soluble sugars (278.19 mg/g), and proteins (9.96 mg/g) (El Semary et al., 2023).

In another study, Abeysiriwardana-Arachchige et al. (2021) developed a three-stage wastewater treatment system utilizing the microalga *Galdieria sulphuraria*, which included biomass generation, hydrothermal liquefaction, and subsequent phosphorus recovery from the resulting biochar. The process yielded high-purity fertilizers struvite (72.7% P) and calcium phosphate (22.7% P) that met US EPA standards, with a total phosphorus recovery efficiency of 95.4% and a production cost of $5.29 per kilogram. These results highlight the multifaceted potential of algae, ranging from enhanced agricultural productivity to effective resource conservation and sustainable phosphorus management (Abeysiriwardana-Arachchige et al., 2021).

In a study by Prisa and Gobbino (2021), it was demonstrated that the application of microbial and algal biostimulants, including effective microorganisms (EM), Trichoderma spp., arbuscular mycorrhizal fungi, and an extract of the brown alga *Ascophyllum nodosum*, significantly improved the agronomic performance of *Aloe barbadensis* Miller. The most pronounced effect was observed with EM treatment, which led to an increase in the number of leaves (20.2 compared to 16.6 in the control), leaf length and width, gel mass, and the concentrations of sugars, fructose, glucose, proline, and aloin in the leaves. In addition, the optical density of the gel was lower in the treated groups, indicating higher product quality. These findings confirm the effectiveness of symbiotic microorganisms and seaweed-based inputs in the organic cultivation of aloe, enhancing both yield and quality while reducing water and fertilizer use (Prisa and Gobbino, 2021).

In a field experiment conducted in Iraq during the spring and autumn seasons, Al-Ghazal et al. (2023) demonstrated that the combined application of a biofertilizer containing a consortium of beneficial microorganisms (*Azospirillum, Azotobacter, Pseudomonas fluorescens, Trichoderma viride, Bacillus* spp.*, Lactobacilli*) and a brown seaweed extract from *Ecklonia maxima* (Algaren-Twin) led to a significant improvement in all major growth and yield parameters of maize (*Zea mays* L.), except for the number of ears per plant. The highest performance was observed with 1000 mL/1000 L of biofertilizer combined with 6 kg/L of seaweed extract, plant height reached 213.1 cm, grain mass per cob was up to 64.9 g, dry biomass yield reached 15.6 t/ha, and total grain yield reached 14.7 t/ha. These improvements were attributed to the nitrogen-fixing, phosphorus-mobilizing, and phytohormone-synthesizing activity of the microorganisms, as well as the bioactive compounds in the seaweed extract that enhanced photosynthesis and metabolism (Aziz et al., 2023).

According to the findings of Pérez-Álvarez et al. (2024), the application of biofertilizers comprising green and red seaweed extracts (*Ulva lactuca*, *Solieria* spp.), Rhizobium sp. bacteria, and the fungus Trichoderma asperellum exerted a multifaceted positive effect on the physiological status and productivity of hybrid maize (*Zea mays* L.). The most pronounced outcomes were recorded with Rhizobium sp. alone (T3), which led to a 13.4% increase in biomass and an 11.82% increase in grain yield compared to the control, along with the highest values for nitrate reductase activity and chlorophyll content (SPAD index +12%). Combined application of all three components (T5) also resulted in a substantial increase in biomass (+11.5%) and yield (+11.6%), enhanced photosynthetic efficiency (total chlorophyll and carotenoid content increased by 16%), and an increase in both leaf number and plant height (by 11.1–11.3%). Treatments with Trichoderma asperellum (T4) and seaweed extracts alone (T2) showed moderate effects, primarily by stimulating the accumulation of photosynthetic pigments, although their impact was less pronounced than in treatments T3 and T5. The authors attribute the effectiveness of Rhizobium sp. to its capacity to fix atmospheric nitrogen, secrete phytohormones (auxins, gibberellins), and solubilize phosphates, thereby improving root development and photosynthetic performance. Notably, the observed reduction in nitrate reductase activity in the combined treatment (T5) did not correspond to a decline in biomass, suggesting an enhanced uptake of ammonium and organic nitrogen forms and indicating a metabolic shift toward more efficient nitrogen assimilation pathways (Baiseitova et al., 2024; Bukharbayeva et al., 2024; Pérez-Álvarez et al., 2024). A summary of representative studies is shown in **Table 2**, while the full dataset is provided **Supplementary Table S1**.

**Table 2:** Representative studies on marine plant extracts used as biofertilizers.

Marine plant	Extract type	Target plant	Observed effects	Reference
*Fucus* sp*iralis*	Seaweed emulsion	Tomato	Stimulated root growth and improved yield	(Mzibra et al., 2021)
*Ulva lactuca*	Purified extract	Hybrid maize (Zea mays)	Increased biomass by 13.4%, enhanced grain yield by 11.8%, and improved chlorophyll content	(Pérez-Álvarez et al., 2024)
*Solieria* spp.	Red seaweed extract	Maize (Zea mays)	Enhanced accumulation of photosynthetic pigments	(Pérez-Álvarez et al., 2024)
*Ascophyllum nodosum*	Commercial biostimulant	Wheat	Improved tolerance to abiotic stress and increased overall yield	(Vigors et al., 2021)
*Sargassum vulgare*	Methanolic extract	Rice	Promoted seed germination and stimulated root elongation	(Al-Khalaifah et al., 2022)

A comparative analysis of recent studies on the use of marine and microalgae in agriculture reveals the high potential of these natural resources as biostimulants for plant growth, sources of essential nutrients, and tools for sustainable soil fertility management. The most significant positive effects have been observed with brown algae such as *Fucus* sp*iralis*, *Ecklonia maxima*, *Ascophyllum nodosum*, and *Cystoseira barbata*, as well as with green and red algae (*Ulva lactuca*, *Solieria* spp.) and cyanobacteria (Phormidium sp.) (Abeysiriwardana-Arachchige et al., 2021; Aziz et al., 2023; Baroud et al., 2024; El Semary et al., 2023; Prisa and Gobbino, 2021). Applications of algae-based extracts and composts have led to substantial improvements in plant growth and biomass accumulation including increased shoot height and leaf and root mass enhanced crop yields (up to +32%) (Gandhi et al., 2024), and elevated levels of photosynthetic pigments (chlorophylls and carotenoids), soluble sugars, and proteins (El Semary et al., 2023; Pérez-Álvarez et al., 2024), in addition, these treatments contribute to increased soil nitrogen, phosphorus, potassium, calcium, and organic carbon content (Gandhi et al., 2024; Verma et al., 2021). The most pronounced effects are achieved through combined applications of seaweeds and microbial agents such as *Rhizobium* sp. and *Trichoderma asperellum* (Pérez-Álvarez et al., 2024).

Particular attention should be given to the use of brown algae in vermicompost production with *Eisenia fetida*, where the incorporation of *Cystoseira barbata* has been shown to reduce concentrations of heavy metals (Zn, Ni, Fe, Pb, Cr, Cu) and enhance the agronomic value of the compost by increasing its content of nitrogen, phosphorus, potassium, calcium carbonate, and organic matter (Türkmen et al., 2021). These findings underscore the relevance of seaweed-derived components not only as nutrient inputs but also as effective agents for bioremediation and ecological soil restoration. Thus, seaweeds are not merely an alternative to synthetic fertilizers, but rather a versatile tool in sustainable agriculture, promoting plant productivity and quality while mitigating the environmental burden on agroecosystems (Prisa and Gobbino, 2021; Türkmen et al., 2021; Verma et al., 2021).

## 3 Aquatic plant extracts in animal agriculture

**Figure 1:**
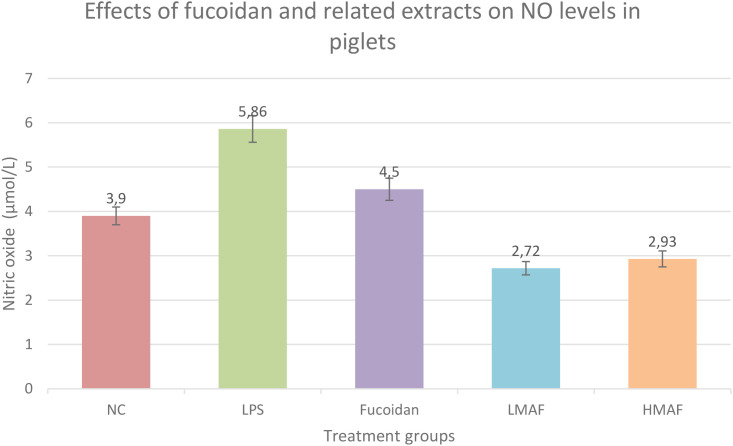
Effects of fucoidan and related extracts on nitric oxide (NO) levels in a model of oxidative stress. Dietary supplementation with fucoidan (300 mg/kg) significantly reduced nitric oxide (NO) levels, serving as a proxy for lipid peroxidation (MDA), and enhanced antioxidant defense through increased superoxide dismutase (SOD) activity. Low-molecular-weight fucoidan hydrolysates (LMAF) showed the most pronounced effect, indicating superior antioxidant and anti-inflammatory activity compared to native fucoidan and high-molecular-weight fractions (HMAF). Data are presented as mean ± SD; p < 0.05 versus LPS group.

Beyond their applications in crop production, algae and their extracts are increasingly recognized in animal husbandry as valuable sources of biologically active compounds with high physiological relevance (the documented effects of marine algae in various animal species, including performance enhancement, immune modulation, and antimicrobial activity, are summarized in **Supplementary Table S2**.) (Rajauria et al., 2015). Historically used as livestock feed in coastal regions, seaweeds are rich in fucoidan, omega-3 fatty acids, vitamins, and essential minerals (Craigie, 2011). Unlike synthetic growth promoters and antibiotics, algal supplements exert multifaceted effects, supporting gut health, modulating immune responses, and enhancing overall productivity, particularly under stress-inducing conditions. As such, their use as functional feed additives is being actively explored as a sustainable alternative to conventional growth enhancers. For instance, supplementation of Ascophyllum nodosum extract in milk replacers for calves has been shown to promote weight gain and reduce the incidence of moderate diarrhea. These effects are attributed to enhanced intestinal barrier integrity and stimulation of innate immune responses (Scaglia et al., 2023). Similarly, the inclusion of *Sargassum*, *Spirulina*, and *Gracilaria* (at 0.5–7.5%) in broiler diets enhanced both cellular and humoral immunity, suppressed Salmonella proliferation, increased levels of lactic acid bacteria (LAB), and reduced intestinal pH, demonstrating the immunomodulatory and antimicrobial potential of algal biomass (Al-Khalaifah et al., 2022). Moreover, certain algal constituents have demonstrated hepatoprotective, antioxidant, and anti-inflammatory activities. Low-molecular-weight fucoidan hydrolysates derived from *Laminaria japonica* have been shown to support intestinal mucosal recovery and modulate the expression of key inflammatory mediators, including TNF-α, IL-1β, IL-6, and nitric oxide. Dietary inclusion of fucoidan (300 mg/kg) in low-birth-weight piglets resulted in a significant reduction in diarrhea incidence, increased superoxide dismutase (SOD) activity, decreased malondialdehyde (MDA) levels, and enhanced hepatic expression of antioxidant genes (Nrf2, CAT, SOD1) and mitochondrial genes (MFN2, SDHA, UQCRB). Under lipopolysaccharide-induced stress, fucoidan activated the Keap1/Nrf2 pathway and preserved mitochondrial function, underscoring its potent antioxidant and hepatoprotective effects in conditions of oxidative stress in piglets (Fu et al., 2024). These effects are illustrated in **Figure 1**.

These findings collectively position marine algae as promising functional ingredients for enhancing animal health and resilience, thereby supporting both productivity and welfare in modern livestock systems (Yin et al., 2024).

In the context of Kazakhstan, which possesses extensive freshwater ecosystems and the Caspian Sea’s coastal waters, particular interest is given to local species of algae and macrophytes, such as *Cladophora, Ulva, Enteromorpha, Spirogyra, Chara*, as well as brown and red algae of the Caspian Basin (*Cystoseira, Gracilaria*) (Jiyenbekov et al., 2019; Sametova et al., 2022). These species are characterized by a high content of biologically active compounds, including polysaccharides (fucoidan, agar, alginates), phenolic compounds, β-carotene, and trace elements. At the same time, given the need for comprehensive metabolic support, especially during the transition period in dairy cattle, it seems promising to expand the range of studied compounds by including marine algae containing metabolically active components such as fucoidan, laminarin, mannitol, and others. In the Kazakh sector of the Caspian Sea, green and diatom algae predominate, with species adapted to relatively low salinity. This research gap highlights the need for comprehensive studies on local algae species, which could serve as a valuable local source of functional feed additives for animals. Their use would reduce dependence on imported ingredients and contribute to the sustainable development of livestock production under regional conditions.

In our previous studies (Amangeldinova et al., 2024, 2025; Yıldırım et al., 2024), we conducted a phytochemical characterization of Rheum extracts obtained via supercritical CO_2_ extraction, revealing a rich composition of polyphenolic compounds, flavonoids, and anthracene derivatives. These findings confirm the potential of plant-based extracts as sources of bioactive substances with antioxidant and hepatoprotective properties. However, considering the need for comprehensive metabolic support, particularly during the transition period in dairy cattle, it appears promising to broaden the range of investigated compounds by incorporating marine algae, which contain metabolically active components such as fucoidan, laminarin, mannitol, and others. Based on the compiled evidence (**Supplementary Table S2**.), marine algal extracts have demonstrated a wide spectrum of physiological, immunological, and metabolic effects across various species of livestock and companion animals. In calves supplemented with *Ascophyllum nodosum* (Scaglia et al., 2023), a reduction in diarrhea incidence and stimulation of innate immunity were observed findings consistent with those seen in studies involving combinations of *Ulva*, *Ascophyllum*, and *Saccharina* (Samarasinghe et al., 2021), where increases in fibrinogen, serum amyloid A (SAA), and haptoglobin levels were recorded. In piglets, fermented fucoidan (LMAF) derived from *Laminaria japonica* (Fu et al., 2024) activated the Keap1/Nrf2 antioxidant pathway and provided mitochondrial protection in liver tissues. Additionally, laminarin from Laminaria digitata (Vigors et al., 2021), enhanced gut colonization by beneficial probiotic genera such as Faecalibacterium and Roseburia, while reducing Campylobacter levels. Supplementation with laminarin and fucoidan elicited both beneficial effects such as elevated SOD activity and decreased MDA levels and potentially adverse outcomes, particularly under prolonged Ascophyllum nodosum exposure in weaned piglets, where elevated expression of pro-inflammatory markers (TNF, CXCL8) was observed (Vigors et al., 2021). In low-birth-weight piglets receiving LMAF (Fu et al., 2024), enhanced expression of antioxidant and mitochondrial genes indicated systemic protection under oxidative stress conditions.

In broiler chickens, supplementation with Sargassum, Spirulina, and Gracil*aria* (Al-Khalaifah et al., 2022) as well as *Palmaria palmata* (Balasubramanian et al., 2021) resulted in improved gut microbiota profiles, including increased levels of lactic acid bacteria, reduced Salmonella and E. coli populations, enhanced body weight gain (BWG), improved feed conversion ratios (FCR), and decreased emissions of volatile gases such as ammonia (NH_3_) and hydrogen sulfide (H_2_S) in excreta. In rabbits (Rossi et al., 2020) a polyphenol-enriched supplement based on *Laminaria* spp. led to a substantial decrease in cholesterol levels (up to −40%) and enhanced meat quality, including improved juiciness, texture, and elevated levels of α-tocopherol and retinol. A particularly novel direction was the use of fermented *Kappaphycus alvarezii* in domestic cats (Mohamad Yusof et al., 2024), which demonstrated high tolerability, improved skin condition (p < 0.05), increased abundance of *Lactobacillus*, and a moderate rise in IgA levels, without adverse effects on body weight, stool quality, or lipid metabolism.

## 4 Biomedical applications

The limited effectiveness and undesirable side effects of synthetic therapeutics used in disease treatment have increased the demand for safer and more effective alternatives (Dehelean et al., 2021). As a result, natural products, especially plant-derived therapeutics, have gained prominence for their ability to address various health problems (Sezer et al., 2024). Among these, marine plants such as seaweed, seagrass, and macroalgae, which are abundant in marine and ocean ecosystems, have attracted considerable attention due to their notable antimicrobial, anticancer, anti-inflammatory, and antioxidant properties (Ribas-Taberner et al., 2025). Marine plants also offer rich pharmacological potential thanks to their high content of secondary metabolites, protective compounds against oxidative stress, generally low toxicity, and unique biological adaptations to environmental challenges. These attributes make them valuable candidates in the search for new natural drug leads (Ameen et al., 2021; Karthikeyan et al., 2022). Numerous studies in literature support the bioactivity of compounds isolated from these organisms (Hamed et al., 2015).

In light of the growing interest in marine plants as sources of pharmacologically active compounds, a detailed table has been compiled to summarize recent studies on their biological activities. The table provides up-to-date information on the anticancer, antimicrobial, antioxidant, and anti-inflammatory properties of marine plant extracts, along with isolated bioactive compounds where available (**Supplementary Table S3**). For each entry, the type of biological activity, the studied marine species, and the compound (if isolated) are presented. Where reported, mechanisms of action are also included to offer deeper insight into their therapeutic potential.

In addition to the catalogued activities, several studies have clarified the mechanistic pathways underlying these bioeffects. For instance, fatty acid-rich extracts of *Melosira nummuloides* suppressed JAK2/STAT3 and MAPK signaling, leading to apoptosis and cell cycle arrest in hepatocellular carcinoma cells (Do et al., 2025). Pigment fractions from Sargassum species triggered apoptosis in colorectal cancer lines while simultaneously demonstrating strong antimicrobial and anti-inflammatory properties. Polysaccharide-rich extracts such as fucoidan and carrageenan were shown to inhibit viral entry by blocking host–pathogen interactions, while phenolic compounds from *Ecklonia* and *Endarachne* species suppressed NF-κB/MAPK signaling, thereby downregulating iNOS and COX-2. These mechanistic insights (Lee et al., 2025), summarized in **Supplementary Table S3**, highlight the ability of marine-derived compounds to act as pathway-specific modulators, offering a rationale for their therapeutic relevance beyond empirical bioactivity testing.

Moreover, recent studies have reported the use of marine plant extracts in the green synthesis of metallic nanoparticles, with particular emphasis on their notable antimicrobial and related biological activities. These nanoparticles represent an eco-friendly and biologically promising alternative for therapeutic applications.

### 4.1 Anticancer applications

Cancer is a major health challenge that affects both physical and mental well-being and ranks as the second leading cause of death worldwide (Wu et al., 2024). According to data from the International Agency for Research on Cancer (IARC), affiliated with the World Health Organization, approximately 20 million new cancer cases were diagnosed globally in 2022, resulting in 9.7 million deaths. GLOBOCAN projections also estimate that new cases of cancer will rise to 35 million by 2050 (Bray Bsc et al., 2024).

In recent years, significant progress has been made in oncology through the development of various treatment strategies, including surgery, radiotherapy, chemotherapy, targeted therapies, and cell-based approaches (Zafar et al., 2025). However, many of these treatments, particularly chemotherapy, also harm healthy cells, leading to serious side effects such as appetite loss, hair loss, and immune suppression. In addition, challenges such as drug resistance continue to hinder treatment success (Anand et al., 2023). Therefore, there is a pressing need for more effective and safer therapies that selectively target cancer cells while minimizing damage to normal tissues (Debela et al., 2021). In light of these limitations, researchers are increasingly turning to alternative and complementary approaches in cancer therapy.

Cancer development is driven by multiple factors, including mutations, disruption of cell cycle regulation, abnormal differentiation, and inhibition of apoptosis (programmed cell death), all of which ultimately lead to uncontrolled cell proliferation and malignant tumor formation (Zhang et al., 2024). Moreover, understanding metastatic spread and tumor biology is essential for identifying new therapeutic targets and improving treatment outcomes (Li et al., 2025).

Thus, natural products have emerged as promising sources for anticancer drug discovery (Deng et al., 2025). Bioactive compounds derived from autotrophic organisms such as microorganisms, plants, animals, and marine plants (especially algae) have shown cytotoxic effects on cancer cells while exhibiting lower toxicity to normal cells (Abuzahrah et al., 2025). In particular, compounds isolated from microalgae, such as polysaccharides, proteins, lipids, carotenoids, and peptides, have demonstrated antitumor activity and chemopreventive potential in various studies (Frazzini and Rossi, 2025). However, research on the biological effects of microalgae-derived compounds remains limited. Among various natural product sources, marine plants such as algae have gained considerable attention due to their unique bioactive constituents and ecological abundance.

**Figure 2:**
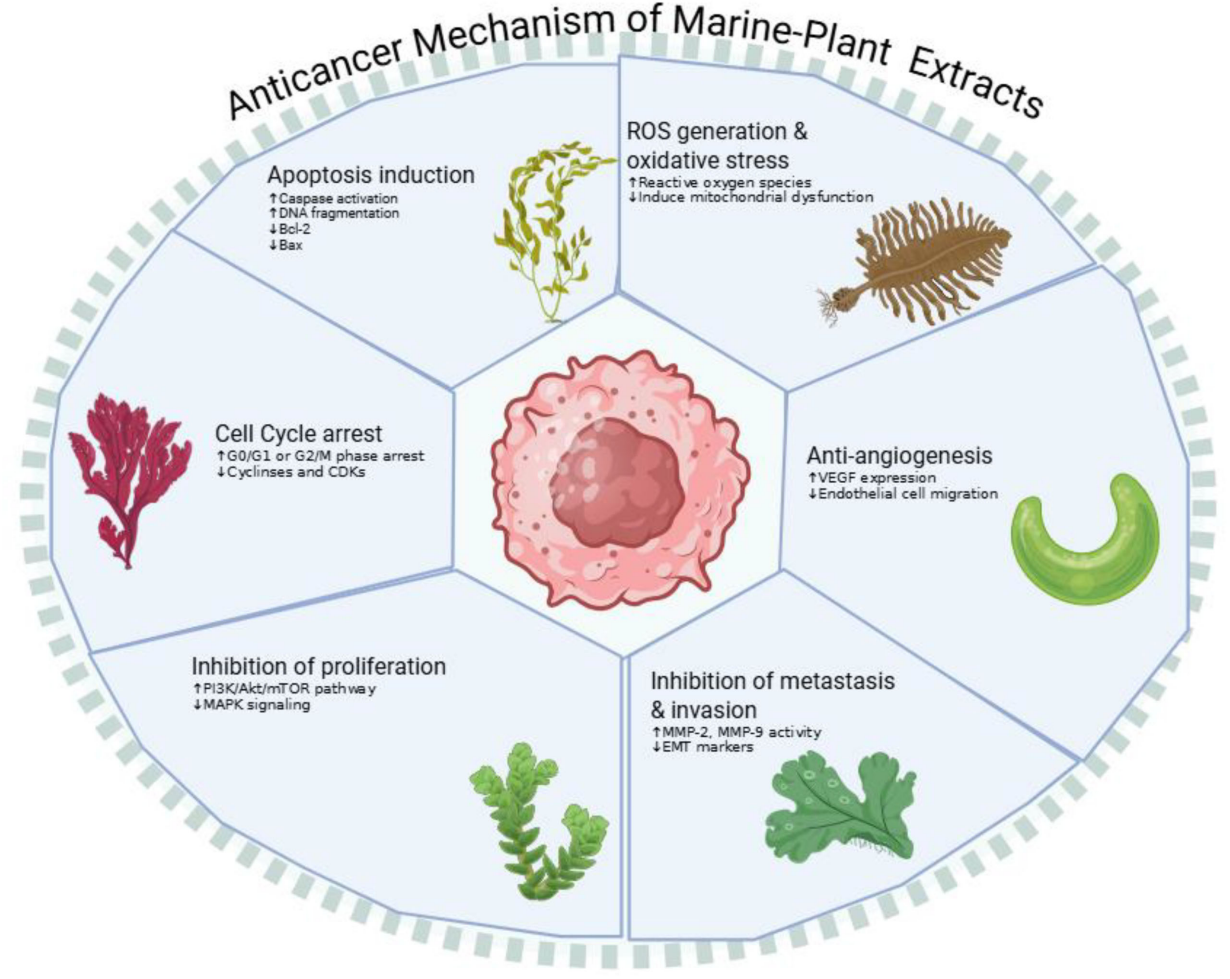
Anticancer action mechanisms of marine plants extracts or isolated compounds.

Recent studies have shed light on the potential of marine-derived extracts and compounds to selectively target cancer cells through multiple mechanisms such as induction of apoptosis, inhibition of cell proliferation, suppression of angiogenesis, and interference with key signaling pathways (**Figure 2**). For instance, the *in vitro* anticancer effects of dolabellane and dolastane derivatives, diterpene compounds derived from *Dictyota dichotoma* brown seaweed, were assessed against DU145, B16F10, HeLa, and MDA-MB231 cell lines. Notably, certain derivatives exhibited significant anticancer activity against B16F10 and DU145 cell lines, with IC_50_ values of 3.53 ± 0.05 and 2.18 ± 0.06 μM, respectively (Ashwini et al., 2025). In another example, PSP-2, a polysaccharide isolated from the edible seaweed *Sargassum pallidum*, showed marked anticancer effects on HepG2 cells by lowering cell viability, promoting apoptosis, enhancing ROS generation, and reducing mitochondrial membrane potential (Wang et al., 2025). Similarly, fucoidan isolated from the brown seaweed *Padina boergesenii* exhibited significant cytotoxicity against HeLa cells, with an IC_50_ value of 38 μg/mL. Apoptotic staining analyses demonstrated that fucoidan prompted apoptosis *via* nuclear fragmentation, G1 phase cell cycle arrest, and elevated ROS production (Cholaraj and Venkatachalam, 2024). Likewise, fucoidan isolated from another seaweed, *Turbinaria decurrens*, showed high activity against HT29 cancer cell line with IC_50_ values ranging from 5.41 to 73.52 μg/mL (Nguyen et al., 2024). The extract of *Thalassia hemprichii* seagrass demonstrated notable anticancer activity against the MCF-7 breast cancer cell line. Molecular docking studies further revealed that betulin, one of the identified compounds, exhibited strong binding affinity toward the cancer-related STAT3 protein (Thirupathi et al., 2025). The extract of the green microalga *Tetraselmis suecica* exhibited dose-dependent anticancer activity in HT-29 (IC_50_: 35 µg/mL) and KB (IC_50_: 20 µg/mL) cancer cell lines, while showing minimal cytotoxicity toward normal HUVEC cells (Asoudeh-Fard et al., 2025). Further emphasizing the broad anticancer potential of marine flora, tests conducted with the extract derived from the marine red algae *Amphiroa anceps* and its liposomal formulation demonstrated a dose-dependent reduction in cell viability in A375 skin cancer cells, with viability decreasing to 16%, and also confirmed the biocompatibility of the formulation (Janani et al., 2025).

The findings above mentioned emphasize that marine-derived bioactive compounds provide diverse chemical scaffolds and mechanisms of action, which could help overcome the limitations of current cancer therapies. A detailed summary of the anticancer activities of extracts obtained from marine plants and the natural compounds isolated from them is presented in the table below. The table outlines the specific types of cancer against which these agents have demonstrated activity, along with the underlying mechanisms through which their anticancer effects are exerted.

### 4.2 Anti-inflammatory and antioxidant applications

Inflammation is the body’s natural defense response to infection, injury, or harmful stimuli, and it plays a vital role in maintaining homeostasis (Chen et al., 2017). Acute inflammatory responses are essential for tissue repair and the elimination of pathogens. However, when these responses become chronic, they can have harmful effects on the body (Soliman and Barreda, 2022). Uncontrolled inflammation is closely linked to various chronic diseases, including cardiovascular conditions, diabetes, neurodegenerative disorders, and especially cancer (Chen et al., 2017). In this context, the excessive production of free radicals, particularly reactive oxygen species (ROS), contributes to tissue damage by inducing oxidative stress and sustaining inflammatory responses. The interaction between inflammation and oxidative stress is bidirectional and forms a central component of the pathophysiology of chronic diseases (Dash et al., 2025).

At the molecular level, specific cellular mechanisms and signaling pathways involved in inflammation play a key role in disease progression (Chen et al., 2017). Antigen-presenting cells such as macrophages and dendritic cells initiate strong immune responses, especially against the lipopolysaccharide (LPS) components of Gram-negative bacteria *via* the TLR4 receptor (De Chiara et al., 2025; Mbongue et al., 2022). This activation triggers the release of proinflammatory cytokines, the overexpression of enzymes such as COX-2, and the production of ROS, all of which contribute to the progression of systemic inflammation (Fischer and Maier, 2015). However, when these mechanisms are excessively or persistently activated, they may lead to serious outcomes such as sepsis, acute lung injury, and even tumor development (Greten and Grivennikov, 2019; Zhao et al., 2021). Therefore, the search for new natural anti-inflammatory agents has become increasingly important. In recent years, natural compounds derived from marine plants have shown promising anti-inflammatory potential by reducing oxidative stress, inhibiting COX-2, and modulating cytokine activity (Li et al., 2021; Vasarri and Degl’Innocenti, 2024).

Numerous studies have demonstrated the promising anti-inflammatory activity of extracts and isolated constituents from marine plants. For instance, an organic extract from the brown alga *Dictyota menstrualis* effectively inhibited neutrophil recruitment to the injury site in a zebrafish larval model of acute inflammation (Obando et al., 2025). Similarly, the immunomodulatory effects of carrageenan and xylan polysaccharides extracted from red seaweeds *Chondrus crispus*, *Ahnfeltiopsis devoniensis*, *Sarcodiotheca gaudichaudii*, and *Palmaria palmata* were evaluated, demonstrating their contribution to wound healing through anti-inflammatory activity (Premarathna et al., 2024).The anti-inflammatory properties of acetone extracts from *Zostera muelleri*, *Halodule uninervis*, *Cymodocea rotundata*, *Syringodium isoetifolium*, and *Thalassia hemprichii* seagrasses, collected from the Great Barrier Reef in Australia, were evaluated. Among them, the extract of *Z. muelleri* significantly reduced the levels of TNF-α, IL-1β, and IL-6 by 29%, 74%, and 90%, respectively, compared to the LPS-treated control group (Perry et al., 2024). Moreover, in the carrageenan (CARR)-induced paw edema model in rats, the sulfated polysaccharide derived from *C. tomentosum* green algae diminished edema and leukocyte migration, preserved redox equilibrium, and exhibited anti-inflammatory properties by modulating various oxidative stress markers at the cellular level (Lakhrem et al., 2024). A sulfated polysaccharide (PSCH) fraction derived from the brown alga *Cystoseira humilis*, consisting of glucuronic acid, arabinose, galacturonic acid, xylose, and fructose, has shown notable anti-edema activity by decreasing malondialdehyde and advanced oxidation protein products in claw tissue (Boujhoud et al., 2024).

Overall, the findings underscore the potential of marine-derived compounds as anti-inflammatory agents. Their structural diversity and capacity to modulate inflammatory and oxidative pathways make algae, seaweeds, and seagrasses promising candidates for novel therapies against chronic inflammation. However, clinical validation and mechanistic studies are needed to fully harness their pharmacological potential.

### 4.3 Antimicrobial application

The indiscriminate use of antibiotics in the treatment of microorganisms that cause infectious diseases has led to the emergence of new resistance mechanisms, including multidrug-resistant bacteria (Ahmed et al., 2024). The World Health Organization identifies antimicrobial resistance as one of the most urgent global health threats, and it is expected to remain a major concern in the coming years (World Health Organization (WHO), 2023). To address this growing concern, it is crucial to discover new drug candidates that act through different antimicrobial mechanisms (Muteeb et al., 2023). In this regard, natural compounds obtained from marine sources, particularly marine plants, have attracted attention due to their inherent antimicrobial properties (Kurhekar, 2020). These approaches may offer valuable alternatives in the global fight against microbial resistance.

Marine-derived natural products offer promising alternatives for bacterial infection treatment. Extracts of the Mediterranean red seaweed *Ganonema farinosum* and brown seaweed *Dictyopteris polypodioides* showed notable antibacterial and antibiofilm activity, particularly against Gram-positive strains (*E. faecalis, S. aureus*) (Ozbil et al., 2025). Lipid extracts from palmitic and linoleic acid-rich seaweeds, including *Hypnea corona*, *Gongolaria barbata*, *Rama rupestris*, and *Undaria pinnatifida*, demonstrated significant efficacy against aquatic *Vibrio* pathogens, particularly antibiotic-resistant strains of *Enterococcus* sp., *Staphylococcus* sp., *Streptococcus agalactiae*, and *P. Aeruginosa* (Stabili et al., 2025). The extract of *Caulerpa cylindracea* seaweed obtained by ultrasound-assisted extraction showed moderate activity against enteric *Morganella morganii* bacteria (Maskur et al., 2025). The extract of *Padina pavonica* L. inhibited biofilm formation by 88–99% and exhibited potent antibacterial activity against *S. aureus*, *E. faecalis*, *P. aeruginosa*, *K. pneumoniae*, and *B. Subtilis* (Makhlof et al., 2024). The green algae extracts obtained from *Enteromorpha* species demonstrated notable antibacterial activity against *P. aeruginosa*, *S. aureus*, and *E. coli* at a concentration of 200 µl/ml, producing inhibition zones of 18 mm, 13 mm, and 18 mm, respectively (Swathi et al., 2024). These findings highlight the therapeutic promise of marine algae-derived extracts in combating resistant bacterial infections.

Beyond antibacterial effects, several marine plant-derived compounds have demonstrated broader antimicrobial activity. For instance, extracts of red and brown algae such *as Gracilaria corticata* and *Sargassum muticum* inhibited the growth of pathogenic fungi including *Candida albicans* and *Aspergillus niger*, suggesting their potential as antifungal agents (Kolanjinathan and Stella, 2011; Younssi Tarhzouti et al., 2024). Sulfated polysaccharides, particularly fucoidan and carrageenan, have been extensively studied for their antiviral properties. Fucoidan isolated from *Undaria pinnatifida* and *Fucus vesiculosus* inhibited influenza A and SARS-CoV-2 entry into host cells, whereas carrageenan fractions from *Chondrus crispus* and *Gigartina skottsbergii* effectively blocked herpes simplex virus (HSV) and human papillomavirus (HPV) attachment (Yang et al., 2023). Moreover, extracts of *Ulva lactuca* exhibited antiprotozoal activity against Leishmania and Plasmodium species (Aruna et al., 2010). Taken together, these findings indicate that marine plant extracts should be considered not only as antibacterial but as broad-spectrum antimicrobial agents, with potential applications in human, veterinary, and agricultural health.

## 5 Marine plants-based metallic nanoparticles and biomedical potentials

**Figure 3:**
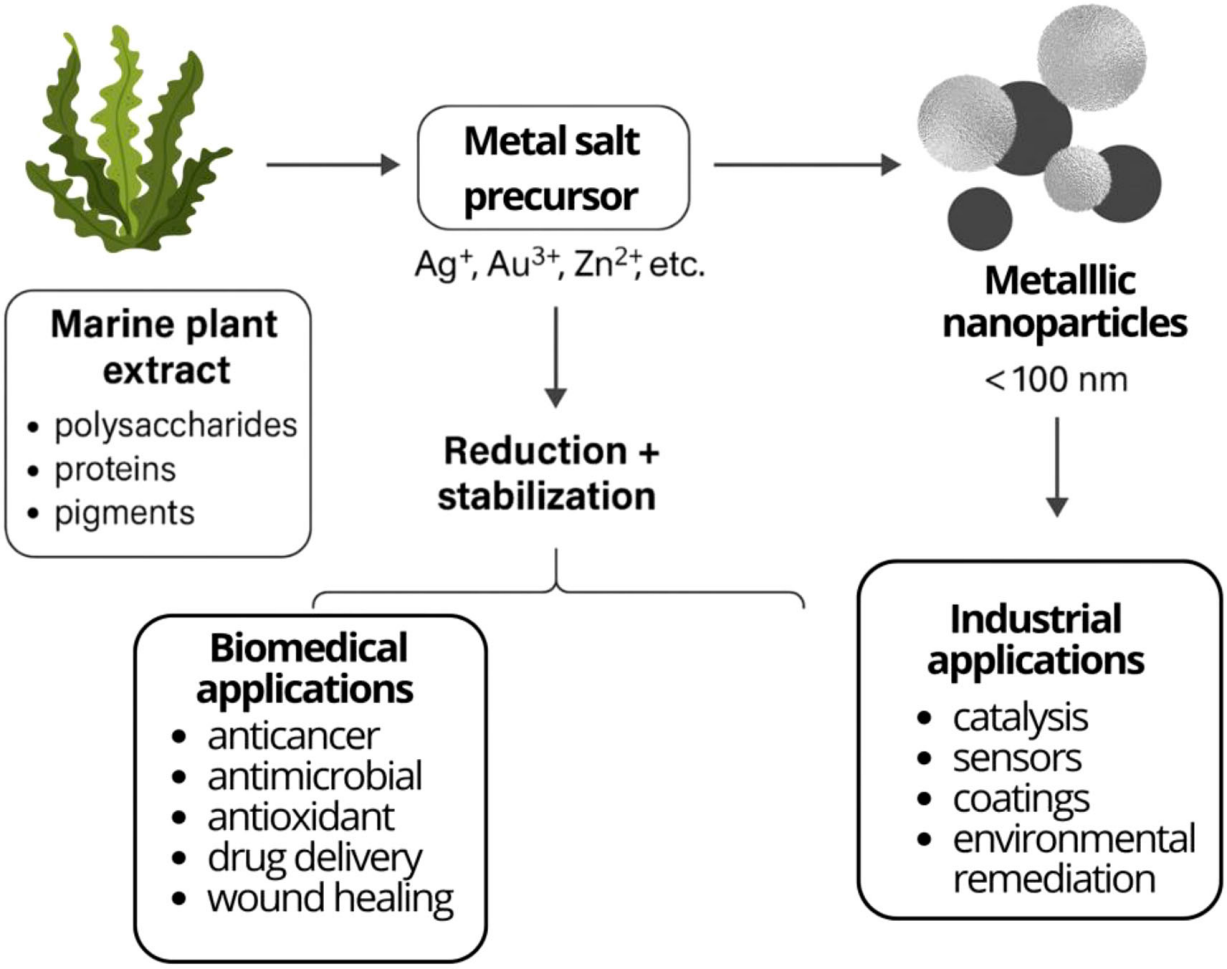
Schematic representation of the green synthesis of metallic nanoparticles using marine plant extracts. Bioactive molecules present in the extracts, including polysaccharides, proteins, and pigments, act as both reducing and stabilizing agents, enabling the conversion of metal salt precursors (e.g., Ag^+^, Au^3+^, Zn^2+^) into stable metallic nanoparticles (<100 nm). The resulting nanoparticles exhibit diverse biomedical applications (anticancer, antimicrobial, antioxidant, drug delivery, wound healing) and industrial applications (catalysis, sensors, coatings, environmental remediation).

Nanotechnology has revolutionized a wide range of scientific and technological fields in recent years, including materials science, biomedicine, environmental science, and agriculture (Malik et al., 2023). Among nanomaterials, metallic nanoparticles (NPs) have gained increasing attention due to their high reactivity and broad functional potential (Aminzai et al., 2024; Burlec et al., 2023; Yıldırım et al., 2023). The side effects associated with synthetic drugs, along with the resulting medical and economic burdens, have led to a growing interest in naturally derived nanoparticles and plant-based therapeutic approaches (Elayaperumal et al., 2025; Karnwal et al., 2024). Plant extracts can be used to produce metallic nanoparticles composed of elements such as gold, silver, zinc, manganese, titanium, platinum, copper and palladium (Aminzai et al., 2024; Pechyen et al., 2024). These nanoparticles are typically smaller than 100 nanometers in diameter and are both environmentally friendly and non-toxic (Gupta et al., 2023). This biological production method, known as green synthesis, offers a sustainable and cost-effective alternative. It does not require hazardous chemicals, elevated temperatures, or high pressures (Morgan and Aboshanab, 2024). Green synthesis can be carried out using various biological materials, including bacteria, fungi, yeasts, viruses, microalgae, and plant extracts (Pechyen et al., 2024). In plant-mediated nanoparticle synthesis, biomolecules such as proteins, polysaccharides, amino acids, enzymes, citrates, and organic acids function as both reducing and stabilizing agents. These components initiate the formation of nanoparticles and contribute to their long-term stability (Adeyemi et al., 2022; Azad et al., 2023). The overall process of green synthesis of metallic nanoparticles using marine plant extracts is illustrated in **Figure 3**.

In this regard, marine plants and macroalgae are recognized as significant biological resources for the synthesis of metallic nanoparticles. Their rich biochemical composition, including polysaccharides, proteins, lipids, phenolic compounds, pigments, and enzymes, enables them to serve effectively in the synthesis process (Teixeira et al., 2025). Furthermore, these bioactive compounds enhance the functionality of the resulting nanoparticles by providing antimicrobial, antioxidant, anticancer, and anti-inflammatory properties (Jeong et al., 2022).

The biological activities of various metallic nanoparticles synthesized from different marine plant species are summarized below.

Silver nanoparticles (GE-AgNPs) synthesized using the red alga *Gracilaria edulis* exhibited notable anticancer activity by inducing apoptosis in HeLa cancer cells (IC_50_: 54.05 μg/mL), while showing minimal cytotoxicity toward healthy HEK293 cells (IC_50_: 83.6 μg/mL) (Ravichandran et al., 2025).

AgNPs synthesized using *Halophila decipiens* extract demonstrated strong antioxidant activity, as evidenced by notable radical scavenging (71.2 ± 2.2% at 25 μg/mL to 37.5 ± 1.5% at 100 μg/mL), nitric oxide scavenging (74.5 ± 1.8% at 100 μg/mL), and hydrogen peroxide scavenging (40.5 ± 2.6%) activities (Roseline et al., 2025).

AgNPs synthesized from *Champia parvula* extract, which is rich in phytochemicals, exhibited strong free radical scavenging capacity and demonstrated the highest antimicrobial activity against *S. mutans* (23 mm), *S. aureus* (21 mm), and *C. albicans* (20 mm). In addition, they showed notable anticancer activity against A549 (IC_50_: 21.54 μg/mL) and HT-29 (IC_50_: 42.36 μg/mL) cancer cell lines (Viswanathan et al., 2024).

AgNPs prepared using *Sargassum* sp. seaweed extract showed a significant anti-inflammatory effect (58.5%) compared to diclofenac sodium (69.2%) (Kavishri et al., 2024).

AgNPs synthesized using extracts of the brown macroalga *Hormophysta triquetra* obtained from different solvents showed significant antibacterial activity against bacterial strains *E. coli*, *B. subtilis*, *S. aureus*, *P. stutzeri* and *P. fragi*. Nanoparticles prepared from aqueous extract (22.5 mM) and ethanolic extract (25 mM) showed high inhibition against *P. fragi*, while those prepared from chloroform-methanol extract (23.5 mM) showed high inhibition against *E. coli* (Bibi et al., 2025).

Iron oxide nanoparticles (Hv-Fe_3_O_4_-NPs) synthesized from the red seaweed *Hypnea valentiae* demonstrated potent anticancer activity against A549 (IC_50_: 66.79 μg/mL) and MDA-MB-231 (IC_50_: 33.89 μg/mL) cancer cell lines. These nanoparticles effectively elevated intracellular ROS levels, thereby inducing apoptosis in MDA-MB-231 cells (Baskar et al., 2024).

Magnetite nanoparticles Fe_3_O_4_ prepared using red algae *Rosenvingea intricata* extract showed dose-dependent cytotoxic effects by inducing apoptosis against Hep3B and PANC1 cancer cell lines (Swathi Pon Sakthi Sri et al., 2024).

TiO_2_ nanoparticles synthesized using the seagrass *Cymodocea serrulata* demonstrated strong antibacterial and antibiofilm activities against methicillin-resistant *Staphylococcus aureus* (33.9 ± 1.7 mm) and *Vibrio cholerae* (36.3 ± 1.9 mm). In addition, antioxidant assays based on TAA (IC_50_: 36.42 μg/mL) and DPPH (IC_50_: 68.85 μg/mL) methods confirmed the nanoparticles’ moderate antioxidant potential. The formulation also exhibited low cytotoxicity in brine shrimp lethality tests (Narayanan et al., 2024).

Another TiO_2_ nanoparticles prepared from *Syringodium isoetifolium* seagrass extract by green synthesis method showed high antibacterial activity (18.6 mmol) against *S. epidermidis* strain and dose-dependent anticancer activity against MCF-7 cell line (IC_50_: 60 μg/mL). The antioxidant effect of the formulation was also proven by different antioxidant tests (DPPH, ABTS and metal chelating) (Sundar et al., 2024).

The antibacterial activities of TiO_2_ NPs prepared using the macroalga *Ulva Lactuca*, when tested against clinical isolates of *E. cloaceae*, *E. avium*, *P. mirabilis*, *P aeruginosa* and *S. pyogenes*, *S. typhi*, *E. faecalis* bacterial strains, showed higher antibacterial activity against clinical isolates (12–19 mm) compared to bacterial strains (9–14 mm). In addition, TiO_2_ NPs exhibited anti-inflammatory effect by inhibiting protein denaturation by 22–78% and showed dose-dependent anticancer activity against oral carcinoma cells (IC_50_ = 28.74 μg/ml) (Mariyam et al., 2025).

Gold nanoparticles (AuNPs) prepared from *Halodule uninervis* extract showed anticancer effects via apoptosis against MDA-MB-231 (IC_50_: 92.59 μg/mL), Capan-2 (IC_50_: 144.4 μg/mL), HCT116 (IC_50_: 89.97 μg/mL) and 22RV1 (IC_50_: 262.03 μg/mL) cancer cell lines (Wehbe et al., 2025).

The antibacterial activities of platinum nanoparticles (PtNPs) prepared using the alga *Tetradesmus obliquus* were investigated against a number of Gram-negative and Gram-positive bacterial strains. Platinum nanoparticles showed remarkable antibacterial activity compared to a reference antibiotic. The inhibition diameters observed in the applied susceptibility tests were as follows: *E. coli* (13 ± 0.20 mm), *V. cholerae* (13 ± 0.30 mm), *P. stuartii* (14 ± 0.21 mm), *S. aureus* (14 ± 0.15 mm), *S. saprophyticus* (13 ± 0.21 mm), *S. pyogenes* (13 ± 0.25 mm) and *S. agalactiae* (12 ± 0.17 mm) Bhilkar, 2025 #134}.

ZnO NPs prepared using *Chlorella vulgaris* extract showed antibacterial activity against *E. coli*, *K. pneumoniae*, *B. subtilis*, and *S. aureus*. Furthermore, the antioxidant activity of the prepared nanoparticles (IC_50_: 19.28μg/mL) was found to be higher than that of the extract (IC_50_: 22.21μg/mL) (Mandal et al., 2025). Copper hydroxide nanoparticles (Cu(OH)_2_ NPs) synthesized using *Coelastrella terrestris* algal extract exhibited strong antibacterial activity against multidrug-resistant *P. aeruginosa* (MIC: 50 µg/mL) bacterial strain and superior antifungal activity against *C. albicans* (MIC: 250 µg/mL) fungal strain when compared with reference antimicrobial agents (Khandelwal et al., 2025). The biological activities and mechanistic pathways of selected marine species are summarized in **Supplementary Table S3**.

## 6 Industrial application

### 6.1 Natural coloring agents

Synthetic pigments are primarily coal tar derivatives obtained as by-products of coal distillation. While many synthetic dyes are controversial due to their potential health risks when used in food and personal care products, their use has been banned in many countries. Some synthetic pigments used in personal care products may contain impurities such as lead acetate, which is toxic to the nervous system. Additionally, some commonly used synthetic pigments are known to be irritants, allergens, or carcinogens. As a result, there is an increasing demand for less toxic natural pigments for use in food products, biotechnological applications, textiles, the energy sector, pharmaceuticals, and personal care items (Azeem et al., 2019).

Marine plants, especially algae, are rich sources of natural pigments. Examples of algae-derived pigments include chlorophyll, β-carotene, fucoxanthin, peridinin, astaxanthin, phycoerythrin, and phycocyanin (Osório et al., 2020). Compared to synthetic pigments, natural pigments are considered more sustainable sources of colorants. In recent years, a significant amount of research has focused on the extraction and purification of natural pigments from marine plants. Below is a brief literature summary of studies conducted in this field:

Seaweeds, which are photosynthetic organisms with high pigment content, are important marine plants due to their content of pigments such as chlorophylls and carotenoids. In a study conducted by Lourenço-Lopes et al., the pigment content of ethanolic extracts obtained by ultrasound-assisted extraction from nine different brown algae (*Ascophyllum nodosum, Bifurcaria bifurcata, Fucus* sp*iralis, Himanthalia elongata, Laminaria saccharina, Laminaria ochroleuca*) collected from the Atlantic coast was determined (Lourenço-Lopes et al., 2022). The HPLC-DAD (High-Performance Liquid Chromatography with Diode Array Detector) method was optimized and used for pigment identification. According to the HPLC results obtained in the study, *L. saccharina* and *U. pinnatifida* were found to contain 10.5-11.5 mg of pigment per gram of dry weight. The three main pigments identified in the algal species were chlorophyll, β-carotene, and fucoxanthin, with fucoxanthin-an important compound for the cosmetics, pharmacology, and food industries-being the most abundant pigment.

A study on seaweeds from the western and southern coasts of Turkey, encompassing brown, red, and green algae (*Cladostephus* sp*ongiosum, Cystoseira foeniculacea, Dictyota dichotoma, Stypopodium schimperi, Colpomenia sinuosa, Petalonia fascia, Hypnea musciformis, Jania rubens, Polysiphonia scopulorum, Caulerpa taxifolia, Ulva rigida, Caulerpa racemosa, Codium fragile*), employed room-temperature ultrasonic extraction to isolate major pigments (Yalçln et al., 2021). The pigments in the extracts were analyzed using a reverse-phase method on an HPLC device.

The extraction of phycoerythrin, a pigment used as a colorant in the food and cosmetic industries, from the red algal species *Pyropia yezoensis* was carried out in a study by Ulagesan et al. in 2021 (Ulagesan et al., 2021). The researchers characterized the isolated phycoerythrin, obtained through various precipitation and purification processes following extraction, using LC-MS/MS and Mascot search, as well as UV-visible and fluorescence spectroscopy.

In the study by Martins et al. (2021), a green method was developed for the simultaneous extraction of chlorophyll and fucoxanthin from the brown alga *Saccharina latissima* using a mixture of 84% ionic liquid and 16% sunflower oil, yielding 4.93 ± 0.22 mg/g chlorophyll and 1956 ± 84 μg/g fucoxanthin, with 82% solvent recovery for reuse (Martins et al., 2021). Similarly, Pereira et al. (2020) extracted phycobiliprotein pigments from the red alga *Gracilaria gracilis* via maceration in phosphate buffer, identifying phycoerythrin as the main pigment, which was purified (purity index = 0.7) and successfully applied as a natural colorant in food products. These studies highlight efficient extraction strategies for high-value algal pigments with potential applications in food and biotechnology (Pereira et al., 2020).

In another study on the extraction of phycobiliprotein pigments from the red alga *Gracilaria gracilis*, Pereira et al. optimized the extraction conditions using both conventional and advanced methods (maceration, ultrasound-assisted extraction via ultrasonic water bath and ultrasonic probe, high pressure-assisted extraction, and freeze-thaw treatment) through Response Surface Methodology (RSM) (Pereira et al., 2020). The yield of phycoerythrin extracted was determined spectroscopically. The researchers reported that maceration was the most effective extraction method for phycoerythrin, achieving a yield of 3.6 mg PE per gram of biomass under the conditions t_1_ = t_2_ = 10 minutes; C = 0.1 M; R = 1:50.

Osorio et al., solvents specific to the type of pigment to be extracted were selected in order to determine the polar and non-polar pigment contents of different algal species, including brown algae (*Himanthalia elongata, Undaria pinnatifida*) and red algae (*Porphyra* spp.) (Osório et al., 2020). The study highlighted that acetone was an effective solvent for the extraction of chlorophylls from both brown and red algae. According to the results obtained, the pigment levels extracted from the red alga *Porphyra* spp. were found to be higher compared to those from brown algae species. In brown algae, the amount of fucoxanthin extracted was reported to be strongly correlated with the total carotenoid content. Among the brown algae species, *Himanthalia elongata* was found to have the highest fucoxanthin-to-total carotenoid ratio.

In a study conducted to evaluate the dyeing efficiency of extracts obtained from certain algal species, Azeem et al. dyed cotton fabrics using aqueous, alkaline, acidic, and organic extracts of brown algae *Iyengaria stellata*, *Sargassum muticum*, *Colpomenia sinuosa*, and the red alga *Laurencia obtusa* under varying conditions of temperature, pH, and exhausting agent concentration (Azeem et al., 2019). Based on the dyeing results, the researchers reported that extraction using 4% KOH and 80% acetone yielded the highest color strength on the dyed cotton fabrics. Among the algal species tested, the highest dye yield was obtained from *L. obtusa*. FTIR analyses conducted to identify the chemical composition of the extracts revealed that the dominant coloring compounds in all four algal species were phenolic in nature.

To determine the optimal extraction conditions and quantify the phycobiliproteins from the red alga *Furcellaria lumbricalis*, Saluri et al. developed a high-performance liquid chromatography (HPLC) method equipped with fluorescence and photodiode array (PDA) detectors (Saluria et al., 2019). Among the phycobiliproteins extracted from this red algal species, the highest yield of R-phycoerythrin was obtained using citrate buffer at pH 6, with a 24-hour extraction at 20 °C (0.13% with fluorescence detector, 0.43% with PDA detector). For the extraction of allophycocyanin, phosphate buffer at pH 6 provided better yields compared to citrate or acetate buffers (0.09% with fluorescence detector, 0.12% with PDA detector).

**Figure 4:**
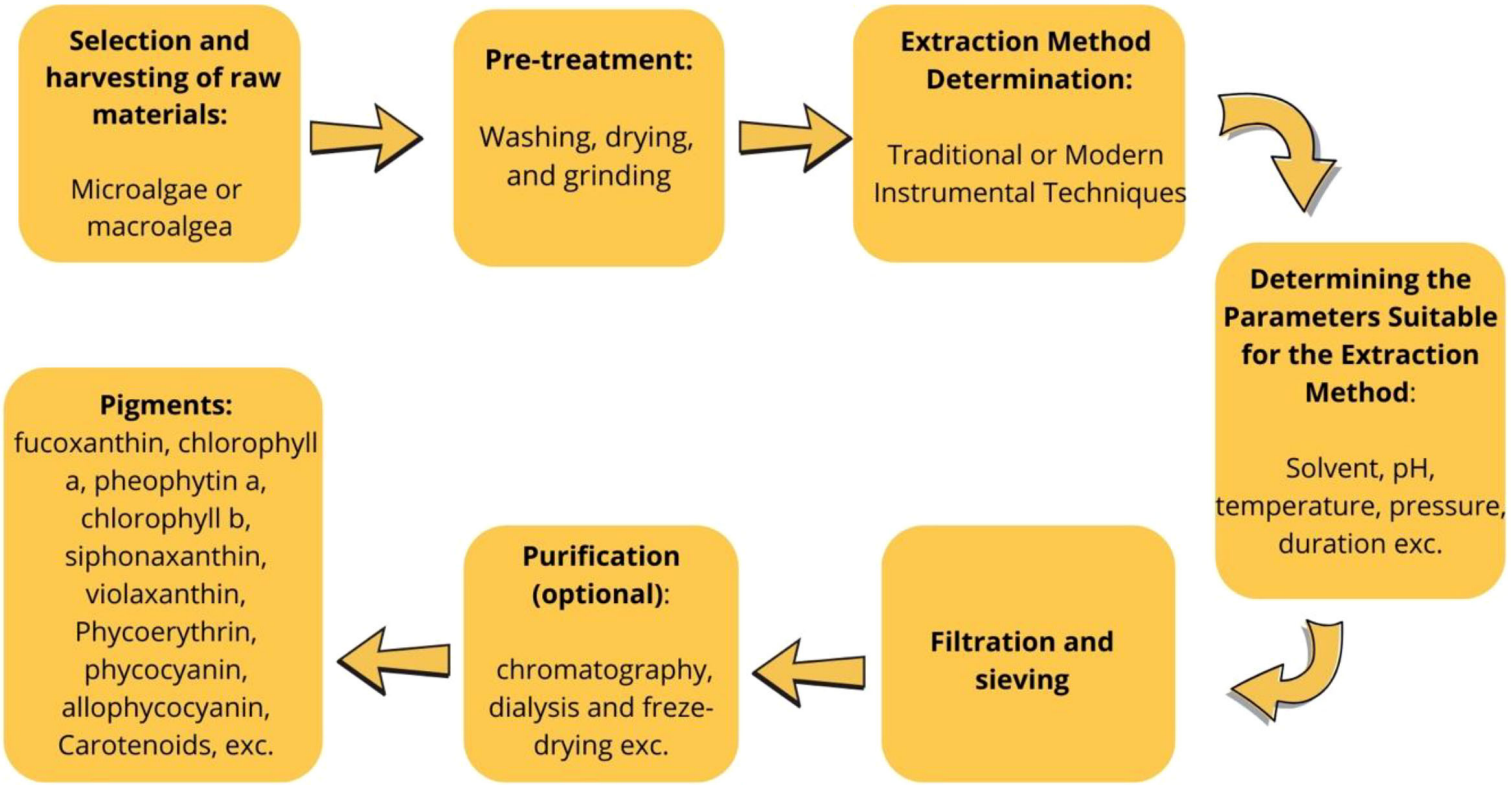
Flow chart illustrating the extraction of pigments from marine plants. The process involves the selection and harvesting of raw materials (microalgae or macroalgae), pre-treatment (washing, drying, and grinding), choice of extraction method (traditional or modern instrumental techniques such as ultrasound-assisted extraction or maceration), optimization of parameters (solvent, pH, temperature, pressure, duration), and optional purification steps (chromatography, dialysis, freeze-drying). Extracted pigments include fucoxanthin, chlorophylls, carotenoids, and phycobiliproteins (phycoerythrin, phycocyanin, allophycocyanin), which serve as sustainable natural colorants for food, cosmetics, and biotechnology.

In a study conducted by Sfriso et al., the extraction of phycobiliprotein (phycoerythrin) from red algal species *Agardhiella subulata*, *Gracilariopsis longissima*, *Gracilaria vermiculophylla*, *Polysiphonia morrowii*, and *Pyropia elongata* was carried out, where a selective extraction method was developed (Sfriso et al., 2018). The intrinsic fluorescence of extracted phycoerythrin was compared with commercial standards. Selective extraction using 1 mM EDTA (pH 9) yielded 95–98%. pH strongly influenced pigment recovery: maximum yields for phycoerythrin, phycocyanin, and allophycocyanin occurred at pH 6–7, but declined at pH 9. Phycoerythrin from *A. subulata* and *P. morrowii* showed distinct properties compared to purified *Pyropia* standards. Representative studies on marine plant pigment extraction and industrial applications are summarized in **Supplementary Table S4**. The overall process of extracting pigments from marine plants using conventional and advanced methods is illustrated in **Figure 4**.

### 6.2 Food packaging

Plastics, which are synthetic polymers derived from petrochemical sources, have high molecular mass. Various chemicals are used during their synthesis to enhance their performance and efficiency. Plastics smaller than 5 mm in size are classified as microplastics, and these pose one of the greatest potential threats to the marine environment worldwide (Laskar and Kumar, 2019).

Microplastics are classified as primary, originating from plastic dust generated during production, and secondary, formed by the degradation of larger plastics. They migrate from rivers to marine environments, contaminating water bodies and entering the food chains of aquatic organisms.

Laskar and Kumar (2019) report that about 5 trillion plastic bags are used annually, with 13 million tons of plastic waste entering aquatic environments. Around 50% of plastics are single-use, and plastic waste accounts for over 10% of global daily waste. As plastic production and consumption rise, posing serious health risks, many countries have enacted laws and policies to reduce their impact. Alongside zero-tolerance measures, research efforts are focused on developing alternative materials to replace plastics (Napper and Thompson, 2023).

Among the most significant efforts are studies investigating the usability of natural extracts for wrapping and preserving food. In this context, there has been a notable increase in recent years in interest toward studies using extracts obtained especially from marine plants. Recent studies have explored the use of algae-derived cellulose for sustainable packaging applications. For instance, Sunesh et al. (2024) extracted micro-sized cellulose filler from *Valoniopsis* pachynema algae collected in the Kanyakumari region of southern India, with the objective of developing bio-based films for food packaging. The extraction process involved alkaline treatment followed by acid hydrolysis (Sunesh et al., 2024). The resulting cellulose was comprehensively characterized using techniques such as X-ray diffraction (XRD), Fourier-transform infrared spectroscopy (FTIR), Raman spectroscopy, thermogravimetric analysis (TGA), atomic force microscopy (AFM), and scanning electron microscopy (SEM). According to the analysis results, the researchers confirmed the semi-crystalline structure with a crystallinity index of 71.9% and a crystallite size of 69.81 nm. In the TGA analysis, they measured the onset degradation temperature at 290 °C and the activation energy at 55.93 kJ/mol. Using AFM, they examined the cellulose surface and determined the surface roughness Ra value to be 33.925 μm.

The potential application of the green seaweed *Ulva rigida* extract, known for its high content of polysaccharides and proteins, in the development of bioplastics for food packaging was investigated by Sonchaeng et al (Sonchaeng et al., 2023). Hot water extraction at 90 °C for 120 min yielded a polysaccharide-rich extract containing glucuronic acid, sulfate, and rhamnose, as confirmed by ^1H NMR, and amino acids (glycine, alanine, glutamic acid, aspartic acid) identified by HPLC. Bioplastic films were prepared by blending the extract with triethyl citrate and glycerol. SEM, XRD, and IR analyses characterized the films, XRD indicated heterogeneous crystallinity and poor compatibility. Glycerol enhanced plasticization, increasing water vapor permeability to 6.5 g·mm/m²·day·kPa and reducing oxygen permeability to <20 mL·mm/m²·day·atm at 30% plasticizer. Mechanical tests showed a fourfold increase in tensile strength and a threefold rise in elongation at break.

In a related study, Cebrián-Lloret et al. (2022) explored the valorization of brown seaweed species *Alaria esculenta*, *Saccharina latissima*, and *Ascophyllum nodosum* through sequential acid and alkaline extraction to isolate alginates, followed by recovery of cellulose-rich residues. These residues underwent further purification steps and were analyzed for their carbohydrate, protein, ash, lipid, lignin, and total phenolic contents. The residues primarily consisted of carbohydrates (35–57%) and proteins (12–37%). Among the species, Alaria and Saccharina yielded higher cellulose content, while Ascophyllum exhibited greater fucoidan concentrations. Self-supporting films were successfully produced from the cellulose fractions of Saccharina and Alaria, exhibiting elastic moduli of approximately 5–7 GPa and elongation at break values ranging from 3% to 5% (Cebrián-Lloret et al., 2022).

Andrade et al. (2021) developed an active coating based on whey protein concentrate incorporated with a 75% ethanolic extract of *Fucus vesiculosus*, aiming to delay lipid oxidation in chicken breast meat. The extract was characterized for its phenolic content and antioxidant activity, and the most potent formulation was integrated into the protein film. The addition of the seaweed extract increased the film’s thickness, tensile strength, and elastic modulus. Furthermore, the coated film effectively inhibited lipid oxidation in chicken breast over a 25-day refrigerated storage period, confirming its potential for use in active food packaging (Andrade et al., 2021).

In the study by Ihamouchen et al. (2020), sodium alginate was extracted from *Dictyopteris polypodioides* through acid and subsequent alkaline treatments. The extracted alginate was blended with polylactic acid (PLA) at different ratios to form biodegradable composite films, with glycerol employed as a plasticizer. FTIR spectroscopy confirmed the presence of uronic acids, while XRD analysis indicated an amorphous polymer structure. The incorporation of alginate into the PLA matrix increased the water vapor permeability of the films, suggesting enhanced moisture sensitivity (Ihamouchen et al., 2020).

de Oliveira et al. (2019) investigated the fabrication of aerogels from the red seaweed *Gelidium sesquipedale* for food packaging applications. An agar-based extract was obtained via ultrasound-assisted extraction at 90 °C for 30 minutes. The extract was rich in phenolics, polysaccharides, proteins, and nanocellulose, as confirmed by analytical assessments. It also exhibited a high aspect ratio (~40) and a crystallinity index of approximately 70%. Hybrid aerogels were then developed by physically cross-linking the extract with cellulose and polyvinyl alcohol (PVA). These aerogels demonstrated significant water retention capacity due to their nanocellulose and hydroxyl group content. Release studies in hydrophilic and hydrophobic food simulants revealed that the aerogels maintained structural integrity and enabled controlled release of bioactive compounds (de Oliveira et al., 2019).

Finally, Arrieta et al. (2018) examined the development of bionanocomposites derived from *Durvillaea antarctica*, a brown algae endemic to Chile, for active food packaging purposes. Extracts were obtained using water, ethanol, and a 1:1 water–ethanol mixture through a 3-hour extraction at 40 °C. The extracts were characterized for total phenolic content and antioxidant activity. Bionanocomposites were produced by combining the extracts with polylactic acid (PLA) and triethyl citrate (TEC). Among the formulations tested, the encapsulation of the extract into electrospun poly(vinyl alcohol) (PVA) fibers and subsequent integration into a PLA matrix yielded superior thermal and mechanical performance, underscoring the potential of this approach for the development of antioxidant-active packaging materials (Arrieta et al., 2018).

### 6.3 Corrosion

Corrosion of metals is a widespread issue encountered during manufacturing, transportation, storage, and utilization, particularly under harsh environmental conditions such as exposure to saline water, oxygen, acidic or basic media, and high humidity. The global annual economic losses directly attributed to metal corrosion are estimated to range from $700 billion to $1 trillion (Sheng et al., 2019). This significant economic burden has intensified the pursuit of effective strategies to protect metal surfaces, especially in industrial applications. Corrosion not only compromises the aesthetic and mechanical integrity of metallic materials but also presents serious safety hazards and can lead to operational disruptions.

Among the most effective approaches for mitigating corrosion are corrosion inhibitors, chemical substances that suppress or delay the degradation of metals through interactions with corrosive agents. Due to their cost-effectiveness, simplicity of application, and broad industrial applicability, corrosion inhibitors have gained widespread use in sectors such as oil and gas extraction, petroleum refining, chemical manufacturing, energy production, and electronics. Their application not only prolongs the service life of metallic components but also significantly reduces maintenance costs.

In line with the principles of green chemistry, which emphasize the minimization of toxic emissions and waste while promoting environmental and human health, there has been a growing interest in developing environmentally benign corrosion inhibitors. In this context, plant-derived extracts have emerged as promising green alternatives to conventional synthetic inhibitors, owing to their natural abundance, biodegradability, and low ecological impact.

#### 6.3.1 Chemisorption

Phytochemicals such as organic acids, esters, terpenes, alkaloids, flavonoids, and tannins, commonly present in plant extracts, often contain functional groups such as carbonyl, amino, hydroxyl, acyl, acetyl, heterocycles, and conjugated double bonds that serve as active adsorption centers (Kokalj, 2022). The adsorption efficiency of these groups is influenced by their electronic configuration, bonding state, and the surrounding electron cloud density. Electron-withdrawing groups, such as carbonyls, tend to attract electrons from adjacent atoms, creating partial positive charges that facilitate their adsorption onto cathodic regions of metal surfaces.

Molecules containing both electron-donating and electron-withdrawing substituents are subject to complex interactions involving inductive, resonance, conjugation, and field effects, all of which, in combination with molecular size and geometry, determine their overall inhibitory performance. Additional factors such as temperature, molecular weight, solubility, concentration of active components, and steric hindrance also play critical roles in modulating corrosion inhibition efficiency.

#### 6.3.2 Physical adsorption

In physical adsorption, the ability of an active compound to donate electrons from its central atom enhances the formation of onium ions (e.g., R–NH_3_^+^) through protonation in acidic environments. These positively charged species can adsorb onto cathodic metal sites via electrostatic interactions (Verma et al., 2022). However, if the central atom is part of an electron-withdrawing moiety, the likelihood of protonation and subsequent adsorption diminishes. This adsorption mechanism is primarily governed by non-covalent intermolecular interactions, including Debye induction forces, Keesom orientation forces, and London dispersion forces. Unlike chemisorption, these interactions are not dictated by the inhibitor’s electron cloud structure but are instead influenced by physicochemical parameters such as atomic radius, polarizability, and dipole moment fluctuations.

#### 6.3.3 Ligand adsorption

Ligand adsorption involves the formation of coordination complexes between donor atoms (e.g., nitrogen or oxygen) within the inhibitor molecule and metal ions or hydrated cations on the metal surface (Kokalj, 2022). The stability and extent of adsorption are influenced by the donor atom’s size, hybridization, and electronic characteristics, as well as by the molecular geometry and steric accessibility of the inhibitor. When steric hindrance is minimal and the coordination bonding energy is low, the resulting chelates are more stable, enhancing the surface coverage and corrosion inhibition efficacy (Verma et al., 2022).

A variety of plant-derived compounds, such as flavonoids, alkaloids, triterpenes, sterols, and fatty acids, have demonstrated corrosion-inhibitory properties, acting as natural defensive agents. Their efficacy, combined with their biodegradability, low toxicity, and sustainability, supports their adoption as green inhibitors in corrosion-prone environments.

#### 6.3.4 Marine plant-derived corrosion inhibitors

In a study published by Rhazzane et al. in 2024, the potential of the seaweed macroalga *Codium tomentosum* to prevent copper corrosion was investigated (Rhazzane et al., 2024). An extract of *Codium tomentosum* obtained by maceration was evaluated as an eco-friendly inhibitor of copper corrosion in 1 M H_2_SO_4_. Gravimetric, electrochemical (polarization, impedance), spectroscopic (FTIR, UV–Vis), and SEM analyses confirmed effective protection, with 82% inhibition at 1 g/L. Efficiency increased with concentration but decreased at higher temperatures. Adsorption was predominantly physical and fitted the Langmuir isotherm.

Additionally, the inhibition mechanism of the extract was studied in detail using quantum chemical calculations and molecular Monte Carlo simulations, and it was stated that these simulations were effective in explaining how the extract binds to the copper surface and how corrosion is inhibited.

Wang et al. investigated the corrosion inhibition effect of seaweed aqueous extract on Q235 carbon steel in a 1 M HCl solution (Q. Wang et al., 2024). In this study, the functional groups present in the seaweed (*Enteromorpha prolifera* leaves) extract were characterized using Fourier Transform Infrared (FTIR) spectroscopy. Corrosion inhibition was evaluated via weight loss, electrochemical tests, SEM, and XPS. The seaweed extract achieved ~95% efficiency for carbon steel in 1 M HCl, acting as a mixed-type inhibitor. Adsorption followed the Langmuir isotherm, and UV-Vis spectroscopy confirmed complex formation between phytochemicals and the steel surface.

In a study conducted by Melyon et al., the effectiveness of different extracts obtained from *Sargassum natans* and *Sargassum fluitans*, invasive brown algae species found in the Guadeloupe region, was investigated as environmentally friendly and innovative corrosion inhibitors for iron in a 1 M hydrochloric acid (HCl) solution (Melyon et al., 2024). Six extraction methods were tested. Ethanol and ethyl acetate extracts showed 72.6% and 70.2% inhibition, respectively, while chloroform cold maceration (SEd) achieved the highest efficiency at 92.0%.

To explain the differences in these results and examine the changes occurring during the corrosion process, the researchers performed SEM and EDX (Energy Dispersive X-ray Spectroscopy) analyses on the SEd extract. Additionally, NMR (Nuclear Magnetic Resonance) analysis was conducted to identify the main chemical components responsible for the inhibitory effect. Structural analysis revealed the presence of fatty acids such as palmitic acid and stearic acid in the extract, and these compounds were identified as the primary agents responsible for the observed anticorrosive activity.

In order to reduce the effects of corrosion, various preventive techniques have been developed, including the incorporation of corrosion-inhibiting additives into protective coatings. In this context, Ibrahim et al. investigated the potential of *Caulerpa lentillifera* (CL) extract as an environmentally friendly corrosion inhibitor in epoxy-based coating systems (Farahin Ibrahim et al., 2024).

Keshk et al., in their study, isolated the compound Fucoidan from the brown algae *Fucus vesiculosus* by interacting it with various solvents and continuously mixing mechanically (Keshk et al., 2024). Fucoidan, characterized by FTIR, UV-Vis, and morphological analyses, inhibited 304 stainless steel corrosion in 3.5% NaCl. Mass loss, EIS, and polarization tests showed 81.7% efficiency at 200 ppm, with decreased double-layer capacitance and slight shifts in corrosion potential. It acted as a mixed-type inhibitor, with adsorption following the Langmuir isotherm via chemical interaction.

The potential of *Chlorella vulgaris* sp. extracts as corrosion inhibitors for API 5L 42 Steel in a 1M HCl acidic environment was evaluated in a study by Almanza et al (Almanza et al., 2024). The researchers obtained two different extracts from the green algae *Chlorella vulgaris* using methanol and a methanol-chloroform mixture at room temperature, and characterized the extracts using FTIR spectroscopy. The effect of the extracts on corrosion inhibition was studied through electrochemical impedance spectroscopy and potentiodynamic polarization. According to the results, significant reductions in corrosion current density and increases in charge transfer resistance were observed: the extract obtained with the methanol-chloroform mixture achieved the highest protection efficiency of 91.20% and a charge transfer resistance of 501.80 Ω at a concentration of 120 ppm. The adsorption of the extracts on the carbon steel surface followed the Langmuir isotherm, and Gibbs free energy calculations indicated a physisorption mechanism for both extracts. Finally, the SEM analysis revealed a reduction in pitting and surface roughness as the inhibitor concentration increased.

Lambert et al. investigated the effect of extracts obtained from *Sargassum fluitans III*, a species of Sargassum found along the coasts of Martinique, on the prevention of corrosion of C38 steel in an acidic medium (1M HCl) (Lambert et al., 2023). The extracts were obtained by performing a 3-hour reflux using water-ethanol mixtures. The researchers characterized the composition of the extracts and, based on phytochemical tests, determined that the extracts contained various chemical groups known to be highly effective against corrosion, such as saponins, steroids, flavonoids, and alkaloids.

The inhibitory effect of the ethanolic extract of *Sargassum muticum* on the corrosion of mild steel in 1 M HCl was investigated by Jeslina et al (Jeslina et al., 2023). It was stated that the inhibition efficiency was evaluated using the weight loss method and potentiodynamic polarization techniques, while the protective film formed on the mild steel surface was examined through the Vickers hardness test.

According to the weight loss measurements obtained in the study, the extract at a concentration of 500 ppm provided 84% efficiency against the corrosion of mild steel. The researchers reported that the adsorption of inhibitor molecules onto the metal surface followed the Langmuir adsorption isotherm, and due to a high correlation coefficient (R² = 0.998), the adsorption was interpreted as monolayer adsorption.

Potentiodynamic polarization results indicated that the extract functioned as a mixed-type inhibitor. In the presence of the inhibitor, the linear polarization resistance increased, while the corrosion current density significantly decreased. According to the researchers, this could be explained by the formation of a protective monolayer film on the metal surface, which blocks anodic and cathodic sites, thereby hindering electron transfer from the metal to the solution.

The inhibitory effect of the red alga *Gelidium* on copper corrosion in 1.0 M sulfuric acid was first investigated by Rhazzane et al (Rhazzane et al., 2023). Red algae samples collected from the El-Jadida region (Morocco) were powdered and ground to a size of 100–60 mesh, then extracted using the maceration method in 1L of 1M sulfuric acid at room temperature.

The results showed that the extract obtained from *Gelidium* effectively inhibited the corrosion of copper when the metal was directly immersed in the corrosive solution. Electrochemical measurements revealed that the inhibitor’s effectiveness increased with concentration, reaching an impressive 93.75% efficiency at 1 M concentration. The researchers determined that the inhibitor was cathodic in nature, functioning by simple adsorption, which blocks the active sites on the copper surface.

The study also found a decrease in inhibitor efficiency as temperature increased, indicating that temperature has an influence on the inhibitor’s behavior. Thermodynamic and kinetic results, when compared, showed that the adsorption is of a physical (physisorption) nature and follows the Langmuir adsorption isotherm. A new corrosion inhibitor derived from *Eucheuma* seaweed for API 5L carbon steel was investigated by Nikitasari et al (Nikitasari. et al., 2022). In this study, *Eucheuma* samples collected from Indonesian seas were extracted using the maceration method in 70% ethanol for 72 hours. The corrosion inhibition performance of the obtained extract was evaluated through electrochemical techniques such as potentiodynamic polarization and electrochemical impedance spectroscopy. Additionally, Fourier Transform Infrared Spectroscopy was employed to identify and confirm the major phenolic compounds present in the extract.

In their study in 2021, Gaber et al. investigated the effectiveness of an inhibitor containing ethanolic extract from *Posidonia oceanica* leaves and polyvinylpyrrolidone (PVP) in protecting mild steel against corrosion in a 1M hydrochloric acid (HCl) solution (Gaber et al., 2021). The inhibitor’s effectiveness was determined through weight loss measurements, open circuit potential, and potentiodynamic polarization results. According to the findings, it was reported that the inhibitor efficiency significantly increased with the extract concentration. At a concentration of 1000 ppm, approximately 81% maximum inhibition efficiency was achieved. FTIR analysis revealed that the extract contained phenolic and polysaccharide compounds, as well as kaolin-like structures, which play a role in corrosion protection. Finally, SEM analysis results showed significant changes in the morphology of the steel surface after treatment with the inhibitor, which was considered as evidence supporting the protective effect of the *P. oceanica*/PVP film layer.

The *Laminaria Japonica* leaf extract, as a mixed inhibitor, was studied for its inhibition effect on Q235 steel in 1 M HCl by Zheng and Wang (Zheng and Wan, 2021). At 200 mg/L, the extract achieved 80% inhibition efficiency, increasing to 88.5% with higher inhibitor concentration and temperature. Adsorption on Q235 steel was physical and conformed to the Langmuir monolayer model. FTIR analysis of the algae extract revealed oxygen- and nitrogen-containing functional groups (–COOH, O–H, C=O, N–H, C–N, C–O). In another study on the inhibitory effect of corrosion control for mild steel, Jeslina et al. used an ethanolic extract of *Sargassum Muticum*, a seaweed, to examine its inhibition effect on the corrosion of mild steel in a 0.5 N HCl solution (Jeslina et al., 2021). Adsorption followed the Langmuir isotherm, and polarization studies classified it as a mixed-type inhibitor. AC impedance confirmed monolayer protective film formation, while AFM and Vickers hardness tests supported surface protection, with hardness values between those of polished and corroded steel.

In a study by Benabbouha et al., the extract obtained from the brown seaweed *Cystoseira baccata* (CBE) via Soxhlet extraction was investigated for its inhibitory effect on the corrosion of carbon steel in a 1M HCl environment (Benabbouha et al., 2020). Inhibitor performance, assessed via weight loss and electrochemical methods, reached a maximum efficiency of 86.5% at 700 mg/L and 25°C (Tafel analysis). Polarization curves indicated mixed-type inhibition, while impedance spectroscopy showed increased charge transfer resistance and reduced double-layer capacitance. Weight loss tests between 25–55°C revealed decreasing efficiency with rising temperature. Adsorption was physical and followed the *Langmuir isotherm*.

*Spirulina*, or blue-green algae, is known not only as a rich source of vitamins and proteins but also for its high antioxidant properties. The inhibitory effectiveness of an extract obtained from *Sunova* sp*irulina* powder was investigated by Jessima et al. against corrosion of mild steel in a 1 M HCl medium (Jessima et al., 2020). Corrosion inhibition studies showed 96% efficiency at 600 ppm and 30 °C. Potentiodynamic polarization confirmed a mixed-type mechanism. Adsorption followed the Langmuir isotherm, indicating initial physisorption with a shift toward chemisorption at higher temperatures. FTIR and SEM analyses characterized the protective film, while UV-visible and fluorescence spectra confirmed Fe^2+^ complex formation with spirulina extract constituents.

Among the marine plant extracts that can be used as effective inhibitors against corrosion in acidic medium for mild steel, *Phyllogorgia dilatata* extract, studied by Fernandes et al., is also included (Fernandes et al., 2020). The researchers obtained the plant extract through the maceration method using a methanol-dichloromethane (1:1) mixture and characterized the extract content using LC-HRMS. They detected the presence of 16 different organic molecules in the extract, including curcumenol, farnesol, curcumol, germacrone, and peridinin. According to electrochemical results, they determined that the extract behaves as a mixed-type inhibitor. As a result of experimental studies, they found that the maximum inhibitor efficiency was achieved at a concentration of 1 g L^-1^ (%93.4). Based on the adsorption data they obtained, they concluded that the adsorption followed the Freundlich adsorption isotherm and confirmed the formation of a protective multilayer film on the surface through SEM/EDX images.

The inhibitory effect of the methanolic extract of the brown seaweed *Sargassum muticum* on the corrosion of carbon steel in a 1 M HCl medium was investigated for the first time by Nadi et al (Nadi et al., 2019). In addition to gravimetric and electrochemical analyses, surface techniques were employed to determine the inhibitory performance of the extract. The researchers reported that the crude methanolic extract of *Sargassum muticum* is rich in the biopolymer alginate. Corrosion evaluation tests revealed that the extract acted as an effective mixed-type corrosion inhibitor, providing an inhibition efficiency of 97% at 30 °C. The researchers also noted that the adsorption of the extract onto the substrate surface followed the Langmuir adsorption isotherm. Furthermore, X-ray photoelectron spectroscopy (XPS) analysis confirmed that the corrosion inhibition mechanism was consistent with a chemical adsorption process.

In a study conducted by Kumar et al. in 2019, the corrosion inhibition effect of *Padina pavonica* marine alga extract on brass in 0.1 N phosphoric acid medium was investigated (Kumar et al., 2019). The researchers calculated thermodynamic and kinetic parameters, determining that the inhibitor acts as a mixed-type inhibitor and follows the Temkin isotherm. Kinetic analysis indicated that adsorption on brass is exothermic, physical, spontaneous, and first-order. Inhibition efficiency rose with extract concentration but declined with temperature, with a maximum of 84.24%.

The corrosion inhibitory effect of the isopropanol extract of the red algae *Halopitys incurvus* on carbon steel in 1 M HCl was investigated in a study conducted by Benabbouha et al (Benabbouha et al., 2018). The researchers reported that the inhibition efficiency increased with increasing concentration and decreased with rising temperature. The highest inhibition efficiency was found to be 81.86% at a concentration of 600 mg/L and a temperature of 25°C. Analysis of the polarization curves showed that the extract acts as a mixed-type inhibitor. Furthermore, the extract’s adsorption on the carbon steel surface was determined to be physical in nature and to follow the Langmuir adsorption isotherm. Details of the literature on the extraction methods of marine plants and their applications in corrosion inhibition are provided in **Supplementary Table S5**.

## 7 Conclusion and future perspectives

Seaweeds and other marine plants are versatile yet underutilized biological resources whose complex biochemical composition, including polysaccharides such as alginate, agar, carrageenan, and ulvan; pigments such as chlorophylls, carotenoids, and phycobiliproteins; phytohormones; antioxidants; polyphenols; and sulfated polysaccharides, confers a broad spectrum of functional properties. In agriculture, these extracts act as biostimulants that promote plant growth, enhance tolerance to abiotic stresses, including drought and salinity, and provide protection against pathogens. In animal husbandry, they serve as functional feed additives that support gastrointestinal health, modulate immune responses, and reduce methane emissions. In environmental management, they contribute to wastewater treatment and bioremediation, enabling nutrient recovery and the generation of valuable by-products.

Beyond their bio- and agronomic applications, marine plant extracts are increasingly valued for industrial uses. Their natural pigments, fucoxanthin, astaxanthin, β-carotene, phycocyanin, and phycoerythrin, offer heat-, UV-, and pH-stable colorants suitable for the food, cosmetic, textile, and ink industries. Polysaccharide-based films and coatings derived from alginate, agar, and carrageenan enable the development of biodegradable, edible, and smart packaging that protects against oxidation, microbial spoilage, and moisture loss. Moreover, phenolics, flavonoids, and sulfated polysaccharides act as eco-friendly corrosion inhibitors, providing biodegradable and non-toxic protection against metal oxidation and biofouling in marine environments, oil and gas infrastructure, and industrial cooling systems.

Despite this breadth of potential, significant barriers hinder large-scale implementation. High cultivation, harvesting, and processing costs, limited infrastructure and technical expertise, and insufficient policy support remain key constraints. Fundamental research is fragmented, with many bioactive compounds, particularly peptides and secondary metabolites encoded by uncharacterized algal gene clusters, still poorly understood. The role of epigenetic regulation in brown algae is only beginning to be explored, and the integration of marine extract bioactivity data, particularly their antioxidant, antibacterial, and anticancer properties, into scalable technologies is still limited. Nanoparticles synthesized via marine extract, mediated green synthesis hold promise as environmentally sustainable platforms for biomedical and industrial applications, yet further research is needed to optimize their performance, scalability, and regulatory acceptance.

Future progress will require coordinated advances in molecular profiling through integrated omics approaches to identify and characterize bioactive compounds, as well as the use of strain improvement techniques to enhance yields and resilience in algal cultures. Developing environmentally friendly extraction methods and circular, cost-effective biorefinery models will be essential to commercial viability. Equally important will be innovations in advanced material design, such as composite films and coatings that combine marine polysaccharides with nanoparticles or other biopolymers to improve functionality, and the integration of marine-derived compounds into nanostructured anti-corrosion systems. By aligning breakthroughs in biotechnology, green processing, materials science, and policy, marine plant extracts can transition from niche research topics to mainstream components of sustainable agriculture, circular-economy industries, and a resilient global bioeconomy.
