# Supplementary material for: Harnessing marine plant extracts for sustainable agriculture, livestock management, industrial use, and biomedical applications
Source: Front Plant Sci. 2025 Nov 6;16:1686511. doi: 10.3389/fpls.2025.1686511 (PMC12630129; doi:10.3389/fpls.2025.1686511)
Supplement: Supplementary file 1 [file Table1.docx]

Supplementary Material

Table S1. Literature review: Extraction of marine plants in biofertilizer applications.

| **Crop** | **Seaweed species** | **Treatment** | **Growth/Biomass** | **Yield** | **Chlorophyll / Pigments** | **Key findings** | **References** |
| --- | --- | --- | --- | --- | --- | --- | --- |
| Pepper (*Capsicum annuum*) | *Fucus spiralis, Bifurcaria bifurcata, Cystoseira gibraltarica* | 1% *F. spiralis* extract | 75.8 cm (shoot); 9.88 g (dry weight) | 7.1 fruits/plant | Brix 5.33; soluble sugars 5.4% | Enhanced vegetative growth, fruit quality, and nutrient content | (Baroud, Tahrouch, and Hatimi 2024) |
| Melon (*Cucumis melo*) | *Phormidium sp.* (cyanobacterium) | Phormidium + Lepidium extract | 96% germination; ↑ fresh biomass | — | Increased chlorophyll, soluble sugars, and proteins | Significant early-stage stimulation and improved seedling vigor | (El Semary et al. 2023) |
| Maize | Ecklonia maxima | 6 kg/L seaweed extract + microbial biofertilizer | Plant height: 213.1 cm | Grain yield: 14.7 t/ha | Increased SPAD index (chlorophyll content) | Highest productivity via synergistic plant-microbe-seaweed action | (Aziz et al. 2023) |
| Aloe | *Ascophyllum nodosum* | EM (effective microorganisms) > seaweed extract | 20.2 leaves/plant; 143.3 g gel mass | - | Increased total sugars, aloin | Improved gel quality and metabolic activity under organic cultivation | (Prisa and Gobbino 2021) |
| Hybrid maize | *Ulva lactuca, Solieria spp.* | T3 Rhizobium / T5 full combination | +13.4% / +11.5% | +11.8% / +11.6% | SPAD increased by 12%; chlorophyll increased by 18.4% | Nitrogen fixation, pigment enhancement, and metabolic efficiency | (Pérez-Álvarez et al. 2024) |
| Moth bean *(Vigna aconitifolia)* | *Ascophyllum nodosum* | 0.10% (root application) | Shoots: 26.2 cm; roots: 17.4 cm | 5 pods/plant | Chlorophyll a: 0.19; Carotenoids: 1.39 mg | Improved photosynthetic activity and root nodulation | (Verma et al. 2021) |
| Maize | *Kappaphycus alvarezii, Gracilaria debilis, Sargassum cinctum* | 7.5% foliar extract | +25% shoot biomass; +59% root biomass | Grain yield: +32%; 3221 kg/ha | Elevated levels of chlorophyll, carotenoids, and antioxidants | Optimal dose identified; enhanced nutrient use efficiency and economic returns | (Gandhi et al. 2024) |

**Table S2.** Effects of marine algae in animal husbandry across different animal species

| № | Algal species | Animal species | Dosage | Observed effects | References |
| --- | --- | --- | --- | --- | --- |
| 1 | *Ascophyllum nodosum* | Calves | 0.3–0.5% in milk replacer | Reduced incidence of diarrhea, improved weight gain, stimulation of the immune system | (Scaglia et al. 2023) |
| 2 | *Sargassum, Spirulina, Gracilaria* | Broilers | 0.5-7.6% | Increased levels of lactic acid bacteria, reduced Salmonella load, lowered intestinal pH, and enhanced humoral and cellular immunity | (Al-Khalaifah, Al-Nasser, and Surrayai 2022) |
| 3 | *Laminaria japonica* (fucoidan, LMAF) | Piglets | 300 mg/kg of feed | Reduction in malondialdehyde levels, enhancement of superoxide dismutase activity, activation of the Keap1/Nrf2 pathway, mitochondrial protection, and upregulation of antioxidant gene expression | (Fu et al. 2024) |
| 4 | *Laminaria digitata* (laminarin), *Ascophyllum nodosum* (fucoidan) | Weaned piglets | Not precisely specified; 35-day trial | Laminarin increased *Faecalibacterium* and *Roseburia* abundance and butyrate levels while reducing Campylobacter; fucoidan increased TNF and CXCL8 expression and decreased CLDN3/5 and OCLN, indicating a pro-inflammatory effect | (Vigors et al. 2021) |
| 5 | *Ulva lactuca*, *Ascophyllum nodosum*, *Saccharina latissimi* | Pre-weaning calves | 50 g/8 L milk (5% DM), 28 days | Increased fibrinogen (SW1, SW2), serum amyloid A (SW2, SW3), and haptoglobin (SW1, SW2); enhanced innate immunity; no significant changes in immunoglobulins (IgG, IgM, IgA) or body weight | (Samarasinghe et al. 2021) |
| 6 | *Laminaria spp.* + polyphenols | Rabbits (lactating females and offspring) | 3 g/kg (low dose, SP1) and 6 g/kg (high dose, SP2) of compound feed, 42 days | Increased carcass weight and dressing percentage, decreased cholesterol levels (up to −40%), improved levels of α-tocopherol and retinol, and enhanced meat texture and juiciness (sensory evaluation) | (Rossi et al. 2020) |
| 7 | *Palmaria palmata* (red alga) | Broilers (Ross 308) | 0.05–0.25% of feed, 42 days | Increased body weight gain and feed conversion efficiency, elevated *Lactobacillus* levels, reduced *E. coli*, increased villus height and width, reduced ammonia and hydrogen sulfide emissions in feces, and improved muscle mass parameters | (Balasubramanian et al. 2021) |
| 8 | *Kappaphycus alvarezii* (fermented) | Domestic cats (healthy) | 2% of body weight, 8 weeks | Improved skin quality (p < 0.05), increased *Lactobacillus* abundance, moderately increased IgA levels; no significant changes in weight, lipid profile, or stool quality; well tolerated and digestible | (Mohamad Yusof et al. 2024) |

**Supplementary Table S3.** Biological activities and mechanistic pathways of selected marine species.

| **Marine Species** | **Active Constituents*** | **Activity** | **Possible mechanism/pathways**** | **Remarks** | **References** |
| --- | --- | --- | --- | --- | --- |
| *Melosira nummuloides* | Fatty acids  (heptadecanoic acid and pentadecanoic acid) | Anticancer  (HepG2, Huh7) | JAK2/STAT3,  MAPK | The Chloroform-Methanol extract of *Melosira nummuloides* arrested the cell cycle and induced apoptosis by suppressing key signaling pathways. | (Do et al. 2025) |
| *Sargassum sp.* | Pigments  Chlorophylls (Chlorophyll-A), Carotenoids (α- carotene), Phycobilliproteins (Phycourobilin) | Anticancer  (Wi38, Caco2)  Antioxidant  Anti-inflammatory  Antimicrobial  (*B. subtilis*, *E. faecalis*,  *S. aureus*, *E. coli*, *S.*  *typhii*, *P. aeruginosa*,  *K. pneumoniae*). | Apoptosis | *Sargassum sp.* acetone extract showed significant anticancer activity (inhibition ratio for Wi38: 174.64 ± 0.5 μg/mL, Caco2: 101.06 ± 0.58).  Its antioxidant activity (IC_50_: 19.65 μg/mL) was lower than that of ascorbic acid (IC_50_: 2.82 μg/mL), the standard antioxidant.  Better anti-inflammatory activity (IC_50_: 4.13 μg/mL) than the reference anti-inflammatory indomethacin (IC_50_: 5.97 μg/mL)  Highest antibacterial activity against *E. faecalis* strain (30 ± 0.25 mm) compared to the reference gentamycin (27 ± 0.20 mm) | (Al-Mur and Alsiary 2025) |
| *Spirulina*  *Chlorella* | Algal proteins | Anticancer  (Caco-2, HT-29, Vero cells)  Antioxidant | Apoptosis | Analysis of proteins extracted *via* deep eutectic solvent revealed that the protein content of *Spirulina* exceeds that of *Chlorella*. The protein content of both algae included the highest levels of glutamic acid, with *Spirulina* exhibiting a greater concentration. According to the ABTS method, the reason for the higher antioxidant effect of *Spirulina* (161.39 mg TEAC/g extract) may be due to its higher tyrosine content compared to *Chlorella* (18.2 TEAC/g extract). Both protein extracts showed significant anticancer activity (IC_50_ for Caco-2: 8.60–9.28 mg/mL and HT-29: 14.16–15.99 mg/mL) and low cytotoxicity against healthy cells. | (Zin et al. 2025) |
| *Gracilaria*  *salicornia* | Alkaloid, steroid/terpenoid, phenolics | Anticancer  (MCF-7) | NI | The anticancer efficacy of the methanol extract of G. salicornia against the MCF-7 cell line was significantly inferior to that of the reference anticancer drug doxorubicin. | (Taba et al. 2025) |
| *Hormophysa cuneiformis* | Phenolics (Gallic acid), flavonoids (Apigenin), tannin (Diphlorethohydroxycarmalol) | Anticancer  (PC-3, HepG-2, Vero cells)  Antioxidant  Anti-inflammatory | NI | *H. cuneiformis* methanolic extract; anticancer activity against PC-3 (IC_50_: 53.31μg/mL) and HepG-2 (IC_50_: 40.80 μg/mL) cells; antioxidant activity: DPPH: %81.14, IC_50_ = 175.31 μg/mL; FRAP: %70.73, IC_50_ = 197.42 μg/mL); COX-2 inhibition: (%74.92, IC₅₀ = 172.81 μg/mL) | (Ahmed, Phycology, and 2025 2025) |
| *Jania rubens* | Polysaccharide, protein, lipid | Anticancer  (HT-29, HCT-116) | Apoptosis induction | All the extract reduced cell viability in a time and dose dependent manner except for lipid extract (only time dependent) and showed strong cytotoxicity. Among the extracts polysaccharide extract was the most anticancer extract in terms of antiproliferative and anti-migratory effects. This may be attributed to its carrageenan content. | (Rifi et al. 2025) |
| *Gracilaria salicornia, Padina boergesenii,*  *and Polycladia myrica* | 1, 2-benzenedicarboxylic acid, diisooctyl ester, hexadecenoic acid, methyl ester | Anticancer  (A375 and Hu02-KP) | NI | *G. salicornia:* cell viability for A375 41-58% and not toxic for healthy cells  *P. boergesenii*: cell mortality for A375 more than 85% and not toxic for healthy cells  *Polycladia Myrica*: no significant anticancer activity or toxicity | (Ramezanpour, Ghanbari Pirbasti, and Waaland 2025) |
| *Chondrus crispus*, *Gracilaria gracilis*, *Porphyra dioica*,  *Pyropia haitanensis*, *Palmaria palmata,* *Ulva rigida* | phenolics, proteins, amino acids, sugars | Anticancer (Caco-2, mouse microglia BV2), antioxidant, anti-inflammatory | NI | No extracts showed any significant cytotoxicity against both cells  *P. dioica*: The most inhibition capacity for lipid peroxidation (IC_50_ = 0.86±0.06 mg/mL.  *P. haitanensis*: The highest aldose reductase (IC_50_ = 0.86 ± 0.06 mg/mL) and 5-LOX inhibition (IC_25_ = 0.26 ± 0.04 mg/mL) | (Trigueros et al. 2025) |
| *Ulva lactuca* | Phenolics (Gallic acid, 4- hydroxybenzoic acid, caffeic acid, vanillic acid, syringic acid, quercetin and kaempferol) | Anticancer (A549), antioxidant, cholinesterase inhibition (AChE, BuChE) | NI | *Ulva Lactuca* extracts showed significant cytotoxicity dose dependent manner.  Total antioxidant activity: 6.272 ± 0.024 mmol/L.  AChE inhibition: 43.82 ± 1.12 μg/mL and 37.66 ± 0.82 μg/mL,  BuChE inhibition: 58.17 ± 1.14 μg/mL and 48.04 ± 0.93 μg/mL. | (Korkmaz 2025) |
| *Caulerpa lentillifera* | Phenolics, flavonoids, chlorophyll a, chlorophyll b | Anticancer (KON, MRC-5)  Antioxidant | Apoptosis, sub-G and G0-G1 cell cycle arrest | Anticancer activity: Acetone extract (IC_40_ = 4.06 ± 176.10, IC_60_ = 297.7 ± 129.90, IC_80_ = 591.3 ± 81.65 μg /mL)  showed the highest inhibitory concentration against KON cells after 24h treatment. All extracts induced the cell death and mitochondrial dysfunction.  Antioxidant activity: Ethanol extract (28.48 ± 1.189) showed higher activity than other extracts. | (Manmuan et al. 2025) |
| *Sargassum cristaefolium* | Daucosterol, Fucosterol, Sitosterol, Stigmasterol | Anticancer (DU145) | *p53*-mediated apoptosis | Induced apoptosis through the generation of oxidative stress and caused cell cycle arrest at the G2/M phase in DU145 cells. Elevated the expression of Bax, p53, and caspase 3 proteins, thereby modulating the intrinsic apoptosis signaling pathway in DU145 prostate cancer cells. *In vivo* studies on BALB/c xenograft model demonstrated anticancer efficacy by inhibiting tumor proliferation and upregulating p53 and caspase 3. | (Sparjan Samuvel et al. 2024) |
| *Gracilaria edulis* | 9-octadecenoic acid methyl ester, hexadecenoic acid methyl ester, 1,2 benzenedicarboxylic acid mono (2-ethylhexyl) ester | Anticancer  (RMS, MCF-7) | Apoptosis (caspase dependent with upregulation of *p21*for RMS and *p53*-mediated for MCF-7 | The increase in Caspase 3/7 activity, DNA fragmentation and p21 gene expression in RMS cells indicate that it plays a key role in the regulation of apoptosis in cells. Apoptosis in MCF-7 cells was mainly achieved by upregulation of p53 gene and compensatory activation of caspase-7 was observed due to partial deletion of CASP-3 gene. Hexadecenoic acid methyl ester and 1,2-benzenedicarboxylic acid mono(2-ethylhexyl) ester compounds, which have the highest relative abundance in the extract, also showed strong binding affinity to p53 and Bcl-2 proteins, confirmed by molecular docking studies, emphasizing the anticancer activity of the extract. | (Gunathilaka et al. 2025) |
| *Posidonia oceanica* | Desmethylverapamil, phenol,2,2’-methylenebis[6-(1,1-dimethylethyl)-4-methyl-,  tris(2,4-di-tert-butylphenyl) phosphate | Anticancer  (MCF-7, HepG2)  Antibacterial (*L.*  *monocytogenes*, *S. aureus*, *B. cereus*,  *S. typhi*, *E. coli*) | NI | Posidonia extract; showed the highest antibacterial activity (21.33 ± 0.28 mm) compared to the reference Gentamicin (27.67 ± 0.91).  Showed higher cytotoxicity than reference anticancer agents Sorafenib, Erlotinib, and Doxorubicin (IC_50_ for MCF-7: 1.75 ± 0.03 mg/mL, HepG2: 1.64 ± 0.06)  Inhibited EGFR kinase enzyme more strongly than reference Erlonitib (114 ± 0.007 mg/mL) (IC_50_ = 95.1 ± 0.006 mg/mL)  Inhibited VEGFR-2 kinase enzyme better than reference Sorafenib (IC_50_ = 85 ± 0.004 mg/mL) (IC_50_ = 26.3 ± 0.001 mg/mL).  Molecular docking studies revealed that Prostaglandin A1-biotin has similar binding affinity to Erlotinib for EGFR enzyme. | (M et al. 2025) |
| *Halodule uninervis* | Emodin | Anticancer  (HepG2, Vero cells) | Apoptosis | Isolated phytoconstituent Emodin; showed significant anticancer activity (IC_50_ = 49.24 μg /mL) and low cytotoxicity on Vero cells (IC_50_ = 654.06 μg /mL).  Induced cell cycle arrest at S phase. In *in vivo* study, emodin reduced the tumor nodules in BALB/c mice. | (Perumal, Arthanari, and Sanniyasi 2025) |
| *Solieria filiformis* | *R*-phycoerythrin | Anticancer  (HCT-116, PC3, SNB-19, HL-60) | NI | Isolated *R*-phycoerythrin compound showed the highest anticancer activity against HL-60 and HCT-116 cell lines. | (de Sousa et al. 2024) |
| *Halimeda tuna* | Stigmasta-5,22-dien-3-ol, 14-Beta-H-Pregna, Hexadecanoic acid, Dodecanoic acid/  Lauric acid, Androst-4-ene-3,17-dione, 1 Docosanol | Anticancer  (HeLa, A549) | NI | The extract showed moderate anticancer activity (IC_50_: 126.460±46.167 μg /mL) against HeLa cell lines compared to Doxorubicin (IC_50_: .647±2.575 μg /mL)  The extract did not show significant anticancer activity (IC_50_: 2771 μg /mL). | (Gazali et al. 2024; Husni et al. 2024) |
| *C. lentillifera* | C180 | Anticancer  (PC-3, LNCaP, 22Rv1) | Apoptosis  inhibition of PSA expression and colony formation | Isolated C180 suppressed the proliferation of PC-3 cells at 5 μg/mL, but complete inhibition was not achieved at 50 μg/mL. | (Wu and Hsiao 2024) |
| *Sargassum echinocarpum* | Fucoidan | Anticancer  (MCF-7) | Apoptosis via lipid peroxidation by increasing MDA levels | Apoptosis induction 24h (49,78%) 48h (72,05%) 72 h (89,35%)  Cytotoxicity against MCF-7 cancer cells 297,58 ± 2,40 ppm concentration  No cytotoxicity against normal TIG 1-20 cells. | (Yunianta et al. 2025) |
| *Turbinaria decurrens* | Fucoidan | Anticancer  (A549) | Inhibited the expression of anti-apoptotic Bcl-2 protein and significantly increased the expression of pro-apoptotic proteins | No cytotoxicity against normal HEK-293 cells.  Cytotoxicity against A549 cells 250 μg /mL fucoidan at 12h, 48h. | (Kumaran et al. 2024) |
| *Caulerpa racemosa* | Caulersin, Caulerpin, Caulerpenyne, Racemosin, Hexadecanamide, Oleamide, Eicosapentaenoic acid, Ageratriol | Anticancer  (NSCLC) | SRC, STAT3, PIK3CA, MAPK1, EGFR, and JAK1 inhibiton | *C. racemosa* extract and isolated Caulersin showed remarkable anticancer activity against NSCLC cancer cells. | (Lau et al. 2024) |
| *Caulerpa lentillifera* | Palmitic acid, tridecanoic acid methyl ester, | Anticancer  (HepG2) | NI | *C. lentillifera* extract showed dose-dependent anticancer activity against HepG2 cancer cells (IC_50_: 1.2 mg /mL). | (Rajasegaran et al. 2024) |
| *Enhalus acoroides*  *Halimeda macroloba* | *E. acoroides* (Alkaloids, steroids, phenolics, flavonoids  *H. macroloba* (Terpenoids, alkaloids, steroids, saponins, phenolics, flavonoids) | Anticancer  (SiHa, HeLa) | NI | *H. macroloba*: IC_50_ for SiHa 17.22 ± 3.93 μg/mL, for HeLa 36.57 ± 7.26 μg/mL  *E. acoroides*: IC_50_ for SiHa 29.67 ± 0.47 μg/mL, for HeLa 54.55 ± 0.64 μg/mL  No cytotoxicity for HEK293 normal cells (>80) | (Navakanitworakul et al. 2024) |
| *Halodule uninervis* | Apigenin, quercetin coumaroyl-glucoside, acacetin, pyromellitic acid | Anticancer  (MDA-MB-231)  Antioxidant | Suppression of the proto-oncogenic STAT3 signaling pathway | Significant antiproliferative effect  DPPH radical scavenging IC_50_: 301.31 μg /mL | (Wehbe et al. 2024) |
| *Posidonia oceanica* | Chicoric acid, *p*-coumaric acid, caftaric acid, trans-cinnamic, rutin hydrate | Anticancer  (LS174)  Antioxidant |  | The extract inhibited the proliferation of LS174 cancer cells.  Insignificant antioxidant activity | (Kevrekidou et al. 2024) |
| *Enhalus acoroides* | Thalassiolin A, Luteolin, luteolin-O-sulphate, Myricetin, di-O-caffeoyl tartaric acid, 6-hydroxy luteolin, *O*-glucoside, Oleamide, Thalassiolin C, O-caffeoyl-O-coumaroyl tartaric acid, betaine | Anticancer  (MCF-7, MDA-MB-231) | Inhibition of HER2/EGFR/HIF-1α pathway | Ethanolic extract anticancer activity: LD50 for MCF-7 220.5650 μg/mL for MDA-MB-231 550.8885 μg/mL  Hexane extract: LD50 for MCF-7 345.9544 μg/mL for MDA-MB-231 1500.6800 μg/mL  Isolated luteolin: LD50 for MCF-7 101.0012 μg/mL for MDA-MB-231 1201.5516 μg/mL | (Prajoko et al. 2024) |
| *Zostera marina* | Zosterabisphenone B | Anticancer  (HCT116) | Increase in caspase-3, caspase-8, caspase-9, PARP and BID protein levels, decrease in Bcl-2 and c-Myc protein levels | No cytotoxicity against HCEC normal cells  Isolated Zosterabisphenone B reduced tumor growth and showed selective cytotoxicity. | (Cacciola et al. 2024) |
| *Thalassia hemprichii*  *Enhalus acoroides* | Chlorogenic acid, caffeic acid, catechin, rutin, quercetin, kaempferol, apigenin at | Anticancer  (MCF-7) | Apoptosis; induce G0/S cell cycle  Increase in caspase 3/7 activity and p53 ve CC2D1A genes  decrease in Bcl-2, Survivin, CDC2 | No cytotoxicity against HSF normal cells  Dose-dependent cytotoxicity against MCF-7 cells | (Mohamed et al. 2024) |
| *Enhalus acoroides* | *p*-hydroxybenzoic acid, rutin, protocathechuic acid, cinnamic acid, gallic acid | Anticancer  (HepG-2) | Decrease in BCL-2, CDC-2 ve CC2DA1 expressions, increase in caspase 3/7 activity | The extract reduced HepG-2 cell growth and showed no cytotoxicity against normal HSF cells. | (Mohamed et al. 2025) |
| *Endarachne binghamiae* | Kansuinin D, acanthoside D, saikosaponin E, bacanoic acid methyl ester, (*R*)-ricinoleic acid | Anti-inflammatory | MAPK (ERK, JNK, p38), IκB | The extract showed its anti-inflammatory effect by inhibiting MAPK and IκB phosphorylation in RAW 264.7 cells and preventing NF-κB from transporting to the nucleus. It also down-regulated the pro-inflammatory cytokines iNOS and COX-2 by regulating the PI3K/AKT signaling pathway. In *in vivo* studies, it inhibited MAPK, NF-κB and PI3K/AKT phosphorylation in mouse lung tissues as *in vitro* studies. | (S. H. Lee et al. 2025) |
| *Sargassum*  *siliquastrum* | Meroditerpenoids (9′-deoxysargachromanol E, 3′,4′-dihydro-4′-hydroxysargachromanol, sargachromanols E, sargachromanols I, apo-9′-sargachromanol I acid | Anti-neuroinflammatory (BV-2 microglial cells) | NI | Except for apo-9′-sargachromanol I, all of the five phytochemicals extracted from algae suppressed NO generation in BV-2 microglial cells (IC_50_ within the range of 8.0–19.5 μM). The highest NO inhibition was shown by 3′,4′-dihydro-4′-hydroxysargachromanol (IC_50_: 8 μM). | (Pan et al. 2025) |
| *Sargassum*  *carpophyllum* | Sulfated polysaccharides | Anti-inflammatory | NI | Extracts of *S. carpophyllum*, derived from acidic, basic, and hot water environments, inhibited nitric oxide production, diminished cellular nitric oxide and reactive oxygen species release, and suppressed pro-inflammatory mediators from RAW 264.7 macrophages, thereby demonstrating anti-inflammatory properties, with the acidic extract exhibiting the most pronounced effect. | (Li et al. 2025) |
| *Dictyota implexa* | Quercetin, tannic acid, phenolic, polysaccharides, agar, fucoidan | Anti- inflammatory, Antioxidant, antibacterial (*E. coli*, *B. cereus*), Anticancer (HT-29, HeLa, Huh7, HepG2, HEK-293), α-amylase | NI | The ethanol extract of the algae showed possible anticancer efficacy against cancer cell lines, but did not exhibit substantial anti-inflammatory, antioxidant, antibacterial, or α-amylase inhibition. | (Trang Thuy and Men 2025) |
| *Rugulopteryx okamurae* | Polyphenols | Anti-inflammatory  Antioxidant | NI | Among the extracts obtained with different extraction methods, Hew 1:20 extract obtained by homogenization using ethanol-water showed higher antioxidant activity compared to other extracts. OAew1:10 extract obtained by shaking overnight showed remarkable anti-inflammatory effect (48.3 ± 7.1% COX-2 inhibition). | (Paulo et al. 2025) |
| *Ecklonia cava, Ecklonia stolonifera, Codium fragile* | Siphonaxanthin, fucoxanthin, dieckol, phlorofucofuroeckol-A | Anti-inflammatory | Inhibits IL-1β expression by modulating the NF-κB/MAPK signaling pathway | The isolated fluorofucofuroecol-A molecule had the most significant anti-inflammatory activity by suppressing the production of inflammatory cytokines, including TNF-α, IL-6, and Il-1β, while diminishing the expression levels of proinflammatory and apoptosis-related genes. | (E. G. Lee et al. 2025) |
| *Padina pavonia*  *Jania rubens* | NI | Immunomodulatory, Anti-inflammatory, Antioxidants, Hepatoprotective | TNF-α and IL-6 significantly reduced, the increase in C-reactive protein (CRP) levels | In in vivo studies with extracts, the extracts increased the number of leukocytes and lymphocytes, while decreasing the rates of neutrophils and monocytes. The extracts notably improved liver damage, reduced serum ALT and AST levels, and effectively diminished necrotic areas, periportal degeneration, fibrosis, and inflammation in the liver. | (Bases et al. 2025) |
| *Devaleraea mollis* | Bioactive hydrolysates, bioactive peptides | Anti-inflammatory  Antidiabetic  Antihypertensive | NI | Biologically active hydrolysates and peptides, protein metabolites isolated from *D. mollis*, shown anti-inflammatory properties by inhibiting DPPIV and ACE1 enzymes while also generating NO and IL6, both of which are anti-inflammatory. | (Mendez et al. 2025) |
| *Sargassum fulvellum* | NI | Anti-inflammatory | Suppressing TNF-α and IL-6 | γ- irradiated water extract reduced NO production compared to non-irradiated water extract of *S. fulvellum.* | (Jo et al. 2024) |
| *Gelidium sesquipedale* | Furyl hydroxymethyl ketone, 3-Methyl-1,2-  cyclopentanedione, 2-Furanmethanol, acetic acid, 1-Hydroxy-2-propanone, Furfural | Anti-inflammatory  Antioxidant  Enzyme inhibition  (AChE, xanthine oxidase, tyrosinase) | 5-LOX inhibition | Subcritical water extract of *Gelidium sesquipedale* residue; IC_50_ for NO 237 ± 38 , Superoxide 41 ± 3, Lipid peroxidation 177 ± 14, 5-LOX 233 ± 30, Xanthine-oxidase 1168 ± 38, Tyrosinase 1148 ± 47 μg/mL | (Trigueros et al. 2024) |
| *Codium fragile* | Alkaloids, saponins, flavonoids, tannins, terpenoids, phytosterol, phenols, cardiac glycosides | Anti-inflammatory  Antioxidant  Antibacterial  (*K. pneumoniae,*  *P. aeruginosa*  *S. aureus, B. cereus*  Antifungal  (*C. albicans*) | NI | The activities of crude methanol extract and polyphenolic (PPF) and polysaccharide fractions (PSF) obtained from this extract were evaluated. DPPH activity for PSF 1685.3 ± 6.5 μg/mL, PPF 1646.3 ± 4.4 μg/mL, CME 5051.1 ± 9.9 μg/mL. CME exhibited the most significant anti-inflammatory activity, while both CME and PSF demonstrated the highest antibacterial efficacy. | (Gangegoda et al. 2024) |
| *Sargassum fusiforme* | Fucoidan | Anti-inflammatory | NI | The fucoidan fraction significantly reduced NO. | (Je et al. 2024) |
| *Sargassum wightii*  *Gracilaria edulis* | Ellagic acid, naringin, carvedilol, bromophenol, salicyclic acid | Anti-inflammatory  Antioxidant  Anticancer  (Caco-2, HepG2) | NI | *S. wightii*: Radical scavenging 75.49 ± 0.12%,  Both extracts showed better anticancer activity than reference Doxorubicin  *G. edulis* showed higher anti-inflammatory effect than standard anti-inflammatory drug compared to *S. wightii.* | (Mohamed et al. 2025) |
| *Phyllophora gibbesii* | Chlorophyll-a, chlorophyll-d, α-carotene, β-carotene, phycocyanin, allophycocyanin, antheraxanthin, β-cryptoxanthin, lutein, violaxanthin | Anti-inflammatory  Antioxidant  Antimicrobial  (*K .pneumonia, E. coli, S. aureus, S. pyogenes, C. albicans*) |  | Radical scavenging: 81.44%, IC_50_: 9.88 μg/mL; ABTS %89.62%, IC_50_: 21.77 μg/mL  Anti-inflammatory activity: 81.25%, IC_50_: 99.75μg/mL  Showed considerable antimicrobial activity against *E. coli* and *C. Albicans.* | (El-Din et al. 2024) |
| *Dictyopteris polypodioides* | Yahazunol, zonarol, isozonarol, 2-fernesyl hydroquinone, zonaroic acid, isozonaroic acid, and chromazonarol, quinone, diacetate, dimethyl ether | Anti-inflammatory | Suppressing iNOS, IL-6, CCL2 expressions | Isolated sesquiterpenoids containing hydroquinone in their structure showed remarkable anti-inflammatory effects. | (Kumagai et al. 2024) |
| *Sargassum latifolium* | Palmitic acid, galactopyranose,6-*O*-(trimethylsilyl)-,cyclic1,2:3,4-bis(methylboronate), heptadecanoic acid, eupatorin, nuatigenin | Anti-inflammatory  Antioxidant | NI | 76,08 % COX-1 inhibition  74,8 % FRAP activity | (Ahmed et al. 2024) |
| *Padina pavonica, Stoechospermum marginatum, Spatolossum macrodontum, Dictyota bartayresiana Turbinaria decurrens* | Fucoidan | Anti-inflammatory  Antiartritis | Suppression of IL-6, IL-1β ve TNF-α expressions  increase in IL-10 and SOD, CAT, GSH, GPX, GST | *T. decurrens*: NO suppression IC₅₀ = 12,93 µg/mL  In *in vivo* studies, showed that fucoidan intake limited inflammation and tissue damage. | (Raj, Gopal, and Sanniyasi 2024) |
| *Alaria esculenta*  *U. lactuca* | Ferulic acid, carnosol,  chlorogenic acid, carnosic acid, pheophorbide, purpurin-18 a, 3'-Hydroxydaidzein | Anti-inflammatory  Antioxidant  Anticancer  (THP-1, HUVEC, HT-29, HepG2) | NI | No cytotoxicity against THP-1, HUVEC, HT-29, HepG2.  Extracts (100 μg/mL) reduced ROS production by 66% and 30%. | (Xavier et al. 2024) |
| *Halimeda tuna* | isolated polysaccharide (PS) | Anti-inflammatory  Antioxidant | NO inhibiton  reduced AOPP and MDA levels, TNF-α  increased SOD and GPx | Isolated PS showed antioxidant, anti-inflammatory activity. | (Kraiem et al. 2024) |
| *Chaetomorpha linum* | Palmitic acid, myrstic acid, oleic acid | Anti-inflammatory | ROS, NO, PGE₂ inhibition and reduce in iNOS, COX-2 expression | The extract's anti-inflammatory properties indicate its promise as a treatment candidate for chronic conditions including atopic dermatitis. | (Frusciante et al. 2024; Osman et al. 2024) |
| *Hormophysa triquetra Padina gymnospora* | Gallic acid, rutin, quercetin, kaempferol, luteolin, hesperidin, catechin, lycopene, zeaxanthin, linalool | Anti-inflammatory  Antioxidant | NO and cytokine inhibiton | Extracts showed antioxidant activity against oxidative stress and anti-inflammatory activity. | (Osman et al. 2024) |
| *Caulerpa racemosa, Digenea simplex Padina pavonica* | Phenolics, flavonoids | Antibacterial (*Yersinia enterocolitica*)  Antioxidant | NI | *P. pavonica* had better antibacterial activity (29.2 ± 0.3 μm) against *Yersinia enterocolitica* compared to other algae and antibiotics examined. The extracts had lower antioxidant activity than the reference antioxidant ascorbic acid, with *P. pavonica* having the maximum activity (34.5 μg/mL). | (Hamad et al. 2025) |
| *Tetraselmis rubens* | 6-Octadecenoic acid, methyl ester, (Z)-, Phytol, 9-Octadecenamide, (Z)-, 2,4-Di-tert-butylphenol | Antibacterial  (*P. corrugata*) | NI | The chloroform extract of *T. Rubens* had the highest antibacterial potency in both *in vivo* and *in vitro* experiments when compared to methanol and ethyl acetate extracts. The zone diameter was 31.33±2.02 mm *in vitro*, whilst *in vivo* investigations with tomato plants demonstrated a significant antibacterial impact, decreasing the incidence of damaged leaves to 5.61%. | (Ouaddi et al. 2025) |
| *Eucheuma cottoni* | Alkaloids, flavonoids, terpenoids, tannins, saponins | Antibacterial (*P. aeruginosa* | NI | The extract exhibited negligible antibacterial activity (13,93 mm) against *P. aeruginosa*. | (Tiarani et al. 2025) |
| *Gelidium*  *amansii* | 2-Bisabolen-1,10,11,12-tetraol from *Aspergillus taichungensis* 299 | Antibacterial (*H. influenzae*, *S. agalactiae*, *S. pneumoniae*) | NI | In comparison to the reference antibiotic chloramphenicol, the isolated sesquiterpene had negligible antibacterial activity, however it demonstrated the best efficacy against the *S. agalactiae* strain (MIC: 8.0 μg/mL). | (Lianlian et al. 2025) |
| *Chlorococcum hypnosporum, Stichococcus bacillaris, Chlorella vulgaris, Chlorolilaea pamvotia, Desmodesmus opoliensis* | NI | Antibacterial (*E. coli, S. aureus, S. typhimurium, E. faecalis*) | NI | The acetone extract of *C. vulgaris* had the most significant antibacterial activity against *B. subtilis* and showed considerable efficacy against *E. coli*. | (Karaca and Soylu 2025) |
| *Fucus vesiculosus, Saccharina latissima* | Fucoidans (Fucose, galactose, xylose, glucose, and mannose) | Antibacterial (*B. subtilis, P. aeruginosa, Proteus mirabilis*) | NI | Isolated fucoidan extracts exhibited an inhibitory zone diameter against the tested bacterial strains. | (Brovko et al. 2025) |
| *Arthrospira maxima* | Spermidine, carnitine, dibutyl phthalate | Antibacterial (Carbapenem-resistant-*K.*  *pneumoniae*) | NI | The algae extract demonstrated antibacterial activity through the disruption of the cell wall (MIC: 500-1000 μg/mL) and also displayed antibiofilm properties. In *in vivo* studies involving mice, the extract demonstrated antibacterial activity comparable to that of the reference antibiotic colistin. | (Selim et al. 2025) |
| *Ulva profunda* | Diethyl phthalate, palmitic acid, (Z)-9-Hexadecenal(cis-9-hexadecenal) | Antibacterial (*Pseudomonas syringae*)  Antioxidant  Anticancer (A549) | NI | Antibacterial activity against *P. syringae* (17,66 ± 0,33 mm)  Antioxidant activity (32,63 ± 1,5 μg/ml)  Anticancer activity for A549 (48,37 ± 1,6 μg/ml) | (Yohannan, Mookkan, and Nagabhushana 2025) |
| *Eucheuma cottoni*  *Sargassum polycystum* | Hexadecanoic acid (CAS) palmitic acid, and heptadecene-(8)-carbonic acid-(1), azulene cyclopentacycloheptene,1,2-benzenedicarboxylic acid, dioctyl ester dioctyl phthalate | Antimalarial (*Plasmodium falciparum*) | NI | *E. cottoni* extract antimalarial activity against *P. falciparum* IC_50_: 11.067 μg/mL  for *S. polycystum* 10.214 μg/mL | (Widiastuti and Kanedi 2025) |
| *Laurencia johnstonii*, *Asparagopsis taxiformis* *Dictyota dichotoma* | NI | Antibacterial (*Clavibacter michiganensis*  , *Ralstonia solanacearum*  *Xanthomonas campestris* (  *Pseudomonas syringae Pseudoxanthomonas*  *sp*.  Antifungal (*Fusarium oxysporum*) | NI | All the extracts inhibited the phytopathogenic strains and *F. oxysporum* fungal strain*.* | (Ana Laura González-Castro. 2024) |
| *Gracilaria edulis*, *Pterocladiella sp*. 4 | *G. edulis* (Palmitic acid, methyl palmitate, eicosane)  *Pterocladiella sp*. 4 (Bis(2-ethylhexyl) phthalate, ethyl palmitate, stearic acid, nonadecanoic acid) | Antibacterial  (*Vibrio harveyi*, *V. parahaemolyticus* *V. alginolyticus)* | NI | Antibacterial activity in the range of 0.625–5 mg/mL | (Kurnia and Kasanah 2025) |
| *Ascophyllum nodosum* | Phlorotannin | Antibacterial  (*P. fluorescens*, *Shewanella putrefaciens*) | Extracellular protease, exopolysaccharide and biofilm inhibition | The extract showed considerable antibacterial activity against food spoilage bacteria *P. fluorescens* and *S. putrefaciens* | (Liu et al. 2025) |
| *Enteromorpha intestinalis, Ulva lactuca, Punctaria plantaginea,*  *Colpomenia sinuosea, Dictyota dichotoma Corallina officinalis* | *n*-hexadecanoic  acid (8) | Antibacterial  (*K. pneumoniae*, *S. aureus*, *S. epidermidis, P. aeruginosa*) | NI | *E. intestinalis* extract was the most effective antibacterial agent. | (Abdel-Tawab, …, and 2025 2025) |
| *Alaria esculenta* | Glucuronic acid, glucose galactose, rhamnose, fucose | Antibacterial  (*E. coli*, *Listeria innocua*) | NI | The extract obtained by ultrasound-assisted extraction showed the highest activity against *E. coli* (MIC: 6.25 mg/mL). | (McGurrin et al. 2025) |
| *Laurencia johnstonii* | Laurinterol | Antibacterial  (*B. altitudinis*, *B. pumilus*, *B. subtilis*, *B. cereus*) | NI | The ethanol extract exhibited the most potent antibacterial effect, while laurinterol, which was isolated from the extract, exhibited significant anti-biofilm activity (>97 and MIC: 3.9 μg/mL) | (Agúndez-Salas et al. 2025) |
| *Macrocystis pyrifera, Undaria pinnatifida* | *Undaria*  (Coumaric acid, gallic acid, rutin, hesperidin)  *Macrocystis*  (Morin, apigenin, Epigallo catechin gallate, taxifolin) | Antibacterial  (*Paenibacillus larvae*) | NI | *Macrocystis* and *Undaria* extracts showed strong antibacterial activity against *P. larvae* strains. (MIC for *Macrocystis* 18.6 μg/mL and for *Undaria* 12.2 μg/mL) | (Fernández et al. 2025) |
| *Corallina officinalis* | Cholesterol, tetramethyl-5'-thymidylic acid, pentadecane, 1-iodotridecane, nonadecane, docosane | Antibacterial  (*S. aureus*, *Citrobacter* sp., *K. pneumoniae*) | NI | Acetone extract had substantial antibacterial efficacy against *K. pneumoniae* (30.2 ± 0.08 mm), although methanol extract exhibited negligible antibacterial activity against *Citrobacter* sp. (15.9 ± 0.08 mm) | (Badr et al. 2025) |
| *Cymodocea serrulata* | NI | Antibacterial  (*E. coli*, *S. mutans*, *S. typhii*, MRSA)  Antioxidant | NI | *C. serrulate* extract showed the highest antibacterial activity against *E. coli* (200 μg/mL-13mm, 100 μg/mL-11mm).  *C. serrulate* extract exhibited significant antioxidant activity (%53.2 ± 2.7) although it showed low antioxidant activity compared to the standard (%68.2 ± 2.2). | (Thiraviyam et al. 2025) |
| *Ulva lactuca*,  *Cystoseira stricta*  *Corallina elongata* | Polyphenols, flavonoids, chlorophyll a, b, caroténoids | Antibacterial  (*B. cereus, B. subtilis,* MRSA *Klebsiella sp*, *S. aureus*)  Antioxidant | NI | *Ulva* extract showed the highest inhibition against *E. coli* (22 mm), *Cystoseria* extract against MRSA (20 mm) and *B. cereus* (19 mm), *Corallina* extract against *B. subtillus* (15 mm). *Pseudomonas* strains showed resistance to all extracts. *C. elongata* extract showed the highest antioxidant activity (IC_50_: 275.70 μg/mL). | (Kherraz-Chemlal et al. 2025) |
| *Enteromorpha intestinalis* | Hexadecanoic acid,  methyl ester, octadecanoic acid,  methyl ester | Anticancer  (HepG-2)  Antioxidant  Antibacterial  (*P. aeruginosa*, *E. coli*, *S. aureus*, *E. faecalis*, *K. pneumoniae, V. fluvialis, V. harveyi*) | NI | Anticancer activity IC_50_: 40.02 ± 3.94 μg/mL  Antioxidant activity: 98.82 ± 1.30 μg/mL, standard Trolox 62.4 ± 0.70 μg/ mL  Antibacterial activity 10 mm against *P. aeruginosa,* 16 mm against *E. coli* | (Al-Mur 2024) |
| *Sargassum polycystum* | Terpenoid, cardiac  glycosides, phenolics, flavonoids | Antioxidant  Antibacterial  (*E. coli*, *S.*  *typhimurium*) | NI | Antibacterial activity: Ethanol extract for *E. coli* 16.50 ± 0.13 mm, methanol extract for *S. typhimurium* 14.6 ± 0.18 mm  Antioxidant activity for methanol extract:  Phosphomolybdenum: 1.96 ± 0.08  DPPH: IC₅₀ = 1.17 mg/mL  ABTS: IC₅₀ = 2.54 mg/mL  Nitric oxide = IC₅₀ = 3.81 mg/mL | (Dip et al. 2024) |
| *Ulva intestinalis* | Alkaloids, flavonoids, glycosides, saponins, terpenoids | Antibacterial  (*P. acnes*, *A. hydrophila*) | NI | Moderate antibacterial activity (at %100 concentration 8.20 mm for *P. acnes* and 7.17 mm for *A. hydrophila*) comparing to reference clindamycin (17.20 mm and 21.18 mm respectively) | (Kurnia and Kasanah 2025) |
| *Sargassum muticum*  *Dictyota dichotoma*  *Cladophora laetevirens*  *Corallina officinalis*  *Ulva lactuca* | Chrysin, vanillin, rutin, kaempferol, cinnamic acid, myricetin, quercetin, hesperetin, naringenin, esculin, benzoic acid | Antibacterial  (*E. coli*, *S. aureus*)  Antioxidant | NI | Antioxidant activity for C. laetevirens with ABTS test 78.65 ± 0.96 μg/mL  *S. muticum* had the best antibacterial activity | (Bouzenad et al. 2024) |
| *Ascophyllum nodosum*  *Palmaria palmata*  *Ulva lactuca* | Polyphenols | Antibacterial  (*E. coli*)  Antioxidant | NI | *A. nodosum* and *U. lactuca* extracts showed significant antibacterial activity against *E. coli*. *A. nodosum* extract also showed the strongest dose-dependent antioxidant effect. | (Hejna et al. 2024) |
| *Halophila stipulacea*  *Halodule uninervis*  *Thalassodendron ciliatum* | Phenolics | Antibacterial  (*B. cereus*, *S. aureus*, *P. aeruginosa*, *E. coli*  Antifungal  (*C. albicans)*  Antioxidant | NI | The extracts demonstrated no significant activity against strains of *E. coli* and *B. cereus*, while exhibiting partial activity against *S. aureus*.  *T. ciliatum* had the highest antioxidant activity (%69.3) | (Payet et al. 2024) |
| *Posidonia oceanica* (L.) | Heptasiloxane, 5-(hydroxymethyl)- 2-Furancarboxaldehyde, 1,3,5-Trisilacyclohexane | Antibacterial  (*E. faecalis* *S. aureus*, *E. coli,* *K. pneumoniae*  and *S. aureus* clinical isolates) | NI | *P. oceanica* extracts showed significant antibacterial activity against *E. faecalis* and *S. aureus* strains, including resistant clinical *S. aureus*, and also showed synergistic effect with ciprofloxacin.  The extract also showed significant antibiofilm activity against *S. aureus* and *E. faecalis* strains. | (Ozbil et al. 2024) |

*****The components with the highest relative abundance within the marine species and the isolated compounds are listed.

** NI: No information

**Table S4.** Literature Review: Extraction of Marine Plants and Their Applications in Colouring Agent

| Entry | Marine plants | Extraction methods and conditions | Colouring agent (s) | References |
| --- | --- | --- | --- | --- |
| 1 | *Ascophyllum nodosum, Bifurcaria bifurcata, Fucus spiralis, Himanthalia elongata, Laminaria saccharina, Laminaria ochroleuca, Pelvetia canaliculata, Sargassum muticum, and Undaria pinnatifida* | Ultrasound-asisted extraction (in 20 ml Ethanol, at 500 W for 55 min) | chlorophyll,  β-carotene, fucoxanthin | (Lourenço-Lopes et al. 2022) |
| 2 | *Cladostephus spongiosum f. verticillatum: C. spongiosum; Cystoseira foeniculacea: C. foeniculacea; Dictyota dichotoma: D. dichotoma; Stypopodium schimperi: S. schimperi; Colpomenia sinuosa: C. sinuosa; Petalonia fascia: P. fascia; Hypnea musciformis: H. musciformis; Jania rubens: J. rubens; Polysiphonia scopulorum: P. scopulorum; Caulerpa taxifolia var. distichophylla: C. taxifolia; Ulva rigida: U. rigida; Caulerpa racemosa var. cylindracea: C. racemosa; Codium fragile: C. fragile* | Ultrasound-asisted extracion (in 5 ml acetone at room temperature for 10 min) | fucoxanthin, chlorophyll a, pheophytin a  chlorophyll b, β-carotene, violaxanthin, siphonaxanthin | (Yalçln et al. 2021) |
| 3 | *Pyropia yezoensis* | Extracted with 80% ammonium  sulfate | phycoerythrin | (Ulagesan, Nam, and Choi 2021) |
| 4 | *Saccharina latissima* (Linnaeus) | Extracted with 84% ionic liquid and 16% sunflower oil mixture | Chlorophyll, fucoxanthin | (Martins et al. 2021) |
| 5 | *Gracilaria gracilis* | Maceration with phosphate buffer (pH: 6.8) at room temperature for 20 min. | phycoerythrin | (Pereira, Barroso, Mendes, and Gil 2020) |
| 6 | *Gracilaria gracilis* | maceration, ultrasound-assisted extraction via ultrasonic water bath and ultrasonic probe, high pressure-assisted extraction, and freeze-thaw treatment | phycoerythrin | (Pereira, Barroso, Mendes, Amaral, et al. 2020) |
| 7 | brown macroalgaes (*Himanthalia elongata, Undaria pinnatifida* and *Laminaria*  *ochroleuca*),  red seaweed (*Porphyra spp.*) | Chlorophylls were extracted  with 100% methanol, 100% methanol acid free, 100% ethanol, 90% acetone and N,N-dimethylformamide  (DMF);  total carotenoids with methanol and methanol acid free;  fucoxanthin (in brown algae) with  DMSO-water (4:1, v/v).  phycoerythrin and phycocyanin from the red seaweed  (Porphyra spp.) were extracted with phosphate buffer  pH 6.8. | Chlorophylls,  Carotenoids,  Fucoxanthin,  Phycoerythrin,  phycocyanin | (Osório et al. 2020) |
| 8 | *Iyengaria stellata*, *Sargassum muticum*, *Colpomenia sinuosa*, *Laurencia obtusa* | Extracted in water bath at 60^o^C for 60 min,  Extracted with organic solvents (ethanol, methanol,  acetone, N-hexane) at 60^o^C for 60 min,  Extracted with alkaline media (NaOH, Na_2_CO_3_, and KOH) at 60^o^C for 60 min in a water bath,  Extracted with acidic (%1-2) media at 60^o^C for 60 min | - | (Azeem et al. 2019) |
| 9 | *Furcellaria lumbricalis* | Extracted in 0.025% NaN_3_ and 50mM pH 6 citrate  buffer at 20 °C in darkness for 24 h | R-phycoerythrin, allophycocyanin | (Saluri, Kaldmäe, and Tuvikene 2019) |
| 10 | *Agardhiella subulata*, *Gracilariopsis longissima*, *Gracilaria vermiculophylla*, *Polysiphonia morrowii*, *Pyropia elongata* | Extracted with 0.1; 1.0; 10 or 100 mM diluted EDTA solution at  different pH ranges from 5 to 9. | phycoerythrin, phycocyanin, allophycocyanin | (Sfriso, Gallo, and Baldi 2018) |

**Supplementary Table S5.** Literature review: Extraction of marine plants and their applications in corrosion inhibition

| Entry | Marine Plant | Extraction Method | Materials and processes applied | Metal | Findings | Ref. |
| --- | --- | --- | --- | --- | --- | --- |
| 1 | *Codium tomentosum* | Maceration (25^o^C for 48 h) | 1 M H_2_SO_4_ solution | Copper | An inhibition efficiency of 82% achieved at a concentration of 1 g/L. | (Rhazzane et al. 2024) |
| 2 | *Enteromorpha prolifera* leaves | Heated deionized water in at 70^o^C for 5 h | 1 M HCl | Q235 Carbon steel | An inhibition efficiency of 95% | (Wang et al. 2024) |
| 3 | *Sargassum natans* and *Sargassum fluitans* | Soxhlet extraction with ethyl acetate, acetone, and ethanol.  Cold successive maceration with chloroform and methanol.  Microwave-assisted extraction with water | 1 M HCl | Iron | Ethanol (72.6%) and ethyl acetate (70.2%) showed significant inhibition efficiency. Highest inhibition efficiency (92.0%) with the extract obtained by cold maceration using chloroform (SEd). | (Melyon et al. 2024) |
| 4 | *Caulerpa lentilifera* | Maceration with ethanol (95%) for 3 days | 3.5 wt% NaCl solution | Epoxy coating SS400 mild steel | Epoxy coatings containing 3 wt% CL extract exhibited approximately 24% higher corrosion resistance compared to the uncoated sample | (Farahin Ibrahim et al. 2024) |
| 5 | *Fucus vesiculosus* | Continuously mixing mechanically extraction with various solvents | 3.5 wt% NaCl solution | 304 SS | Maximum efficiency of 81.7% was achieved at a concentration of 200 ppm | (Keshk et al. 2024) |
| 6 | *Chlorella vulgaris* sp. | Maceration with methanol and methanol-chloroform at room temperature | 1 M HCl | API 5L 42 Steel | Achieved the highest protection efficiency of 91.20% and a charge transfer resistance of 501.80 Ω at a concentration of 120 ppm of methanol-chloroform mixture. | (Almanza et al. 2024) |
| 7 | *Sargassum fluitans III* | Refluxed with etnaol-water for 3h | 1 M HCl | C38 steel | An inhibition efficiency of 88% | (Lambert et al. 2023) |
| 8 | *Sargassum muticum* | Maceration with ethanol at room temperature for 8 h | 1 M HCI | Mild steel | 500 ppm extract concentration provided 84% efficiency against the corrosion of mild steel | (Jeslina et al. 2023) |
| 9 | red alga *Gelidium* | Maceration with 1M H_2_SO_4_ at room temperature | 1 M H_2_SO_4_ | Copper | 93.75% efficiency at 1 M concentration | (Rhazzane et al. 2023) |
| 10 | *Eucheuma* | Maceration with 70% ethanol for 72 h | 0.1 M HCl | API 5L carbon steel | A high inhibition efficiency of 96.4% at 500 ppm concentration | (A. Nikitasari. et al. 2022) |
| 11 | *Posidonia oceanica* | Refluxed with % 70 ethanol at 75 ^o^C for 7h | 1 M HCl | Mild steel | A high inhibition efficiency of 81% at 1000 ppm concentration | (Gaber et al. 2021) |
| 12 | *Laminaria Japonica* | Maceration with deionize water at room temperature | 1 M HCl | Q235 steel | A concentration of 200 mg/L, the inhibition efficiency of the extract reached up to 80%. | (Zheng and Wan 2021) |
| 13 | *Sargassum Muticum* | Refluxed with ethanol for 8h | 0.5 M HCl | Mild steel | A high inhibition efficiency of 99% at 500 ppm concentration | (Jeslina et al. 2021) |
| 14 | *Cystoseira baccata* | Soxhlett extraction with isopropanol (70%) for 8 h | 1 M HCl | Carbon steel | Maximum inhibition efficiency was found to be 86.5% at a concentration of 700 mg/l at 25°C. | (Benabbouha et al. 2020) |
| 15 | *Sunova spirulina* | Refluxed with 1 M HCl at 80^o^C for 3 h | 1 M HCl | Mild steel | an inhibition efficiency of 96% at a 600 ppm concentration at 30 ^o^C | (Jessima, Subhashini, and Arulraj 2020) |
| 16 | *Phyllogorgia dilatata* | Maceration with methanol:dichloromethane (1:1) for 6 h | 1 M HCl | Mild steel | Maximum inhibitor efficiency was achieved at a concentration of 1 g L^-1^ (%93.4) | (Fernandes et al. 2020) |
| 17 | *Sargassum muticum* | Refluxed with methanol for 6 h | 1 M HCl | Carbon steel | An inhibition efficiency of 97% at 30 °C | (Nadi et al. 2019) |
| 18 | *Padina pavonica* | Soxhlet extraction with ethanol | 0.1 N H_3_PO_4_ | Brass | The maximum inhibition efficiency was determined to be 84.24%. | (Kumar et al. 2019) |
| 19 | *Halopitys incurvus* | Soxhlet extraction with 70% isopropanol for 8 h | 1 M HCl | Carbon steel | The highest inhibition efficiency was found to be 81.86% at a concentration of 600 mg/L and a temperature of 25°C | (Benabbouha et al. 2018) |

Reference

A. Nikitasari., G. Priyotomo., A. Royani., and S. Sundjono. 2022. “Exploration of Eucheuma Seaweed Algae Extract as a Novel Green Corrosion Inhibitor for API 5L Carbon Steel in Hydrochlorid Acid Medium.” *International Journal of Engineering, Transactions B: Applications* 35(6):1209–16. doi:10.5829/ije.2022.35.06c.13.

Abdel-Tawab, AM, … S. Salah-Egyptian Journal of, and undefined 2025. 2025. “Comparative Antimicrobial Activities of Some Mediterranean Sea Seaweeds-Derived Extracts.” *Ejchem.Journals.Ekb.EgAM Abdel-Tawab, S Salah, AE Elshehry, HAH Ibrahiem, HML El Gamal, HEB GhonamEgyptian Journal of Chemistry, 2025•ejchem.Journals.Ekb.Eg*. https://ejchem.journals.ekb.eg/article_382799.html.

Agúndez-Salas, Martha Patricia, Ruth Noemí Aguila-Ramírez, Ana Laura González-Castro, Sara García-Davis, and Mauricio Muñoz-Ochoa. 2025. “Unraveling the Anti-Biofilm Properties of Laurinterol on Pioneer Biofouling Bacteria from the Red Seaweed Laurencia Johnstonii.” *Journal of Applied Phycology* 37(2):1357–62. doi:10.1007/S10811-024-03439-Z/METRICS.

Ahmed, SI, FMA Akl-Egyptian Journal of Phycology, and undefined 2025. 2025. “In Vitro Potential of Hormophysa Cuneiformis Methanolic Extract as Antioxidant, Anti-Inflammatory, Anticoagulant, and Anticancer.” *Journals.Ekb.EgSI Ahmed, FMA AklEgyptian Journal of Phycology, 2025•journals.Ekb.Eg*. https://journals.ekb.eg/article_409016.html.

Ahmed, Suzan I., Mostafa M. El-Sheekh, Faiza M. A. Akl, Mofida E. M. Makhlof, and Sahar E. Abo-Neima. 2024. “The Brown Seaweed Sargassum Latifolium Combined with Low-Level Laser Irradiation Reduces Hypercholesterolemia in Rats by Alleviating Oxidative Stress and Inflammation.” *South African Journal of Botany* 172:686–700. doi:10.1016/J.SAJB.2024.07.056.

Al-Khalaifah, Hanan S., A. Al-Nasser, and T. Surrayai. 2022. “Effects From Dietary Addition of Sargassum Sp., Spirulina Sp., or Gracilaria Sp. Powder on Immune Status in Broiler Chickens.” *Frontiers in Veterinary Science* 9:928235. doi:10.3389/FVETS.2022.928235/BIBTEX.

Almanza, Edgar, Lizeth Del Carmen Gutierrez Pua, Yaneth Pineda, Wilson Rozo, Mauricio Marquez, and Ana Fonseca. 2024. “Eco-Friendly Chlorella Vulgaris Extracts for Corrosion Protection of Steel in Acidic Environments.” *Heliyon* 10(21). doi:10.1016/J.HELIYON.2024.E39717/ASSET/E020D14E-7695-4B35-901D-ED9D9144B76A/MAIN.ASSETS/GR10.JPG.

Al-Mur, Bandar A. 2024. “In Vitro Anticancer, Antioxidant and Antibacterial Activities of Crude Extract Prepared from Enteromorpha Intestinalis Habited in Jeddah, Saudi Arabia.” *Saudi Journal of Biological Sciences* 31(7):104026. doi:10.1016/J.SJBS.2024.104026.

Al-Mur, Bandar A., and Waleed A. Alsiary. 2025. “Bioactivities Related to Pigments Content of Sargassum Sp. Collected from Jeddah Coast, Saudi Arabia, Red Sea.” *Egyptian Journal of Aquatic Research* 51(2):181–88. doi:10.1016/J.EJAR.2025.02.001.

Ana Laura González-Castro. 2024. “Vista de Evaluation of Some Seaweed Extracts from Baja Peninsula, Mexico, against Plant Pathogens.” https://hidrobiologica.izt.uam.mx/hidrobiologica/index.php/revHidro/article/view/1785/1253.

Azeem, Muhammad, Naeem Iqbal, Riffat Ayesha Mir, Shahid Adeel, Fatima Batool, Ali Ahmad Khan, and Sadaf Gul. 2019. “Harnessing Natural Colorants from Algal Species for Fabric Dyeing: A Sustainable Eco-Friendly Approach for Textile Processing.” *Journal of Applied Phycology* 31(6):3941–48. doi:10.1007/S10811-019-01848-Z/METRICS.

Aziz, Moyassar Mohammed, Waleed Al-Juheishy, Salim Abdullah, Younis Al-Ghazal, Mohammed Aziz, Waleed Khalid, and Shahatha Al-Juheiehy. 2023. “Response of Growth and Yield of Corn (Zea Mays L.) to Bio-Fertilizer and Sea-Algae Extract.” *International Journal of Agricultural and Statistical Sciences. Int. J. Agricult. Stat. Sci* 19(1):161–65. doi:10.59467/IJASS.2023.19.161.

Badr, HA, AE Abo-Amer, MA Abu-Gharbia, and DH Ibrahim. 2025. “Antibacterial Activity of Corallina Officinalis Seaweed Extracts Against Some Pathogenic Bacteria.” *Sohag Journal of Sciences*. https://sjsci.journals.ekb.eg/article_418762.html.

Balasubramanian, Balamuralikrishnan, Sureshkumar Shanmugam, Sungkwon Park, Neeraja Recharla, Jin Su Koo, Ines Andretta, and In Ho Kim. 2021. “Supplemental Impact of Marine Red Seaweed (Halymenia Palmata) on the Growth Performance, Total Tract Nutrient Digestibility, Blood Profiles, Intestine Histomorphology, Meat Quality, Fecal Gas Emission, and Microbial Counts in Broilers.” *Animals 2021, Vol. 11, Page 1244* 11(5):1244. doi:10.3390/ANI11051244.

Baroud, Said, Saida Tahrouch, and Abdelhakim Hatimi. 2024. “Effect of Brown Algae as Biofertilizer Materials on Pepper (Capsicum Annuum) Growth, Yield, and Fruit Quality.” *Asian Journal of Agriculture* 8(1):25–31. doi:10.13057/ASIANJAGRIC/G080104.

Bases, Eman, Mostafa M. El-Sheekh, Shimaa M. El Shafay, Rania El-shenody, and Mohamed Nassef. 2025. “Therapeutic Anti-Inflammatory Immune Potentials of Some Seaweeds Extracts on Chemically Induced Liver Injury in Mice.” *Scientific Reports 2025 15:1* 15(1):1–15. doi:10.1038/s41598-025-87379-9.

Benabbouha, T., R. Nmila, M. Siniti, K. Chefira, H. El Attari, and H. Rchid. 2020. “The Brown Algae Cystoseira Baccata Extract as a Friendly Corrosion Inhibitor on Carbon Steel in Acidic Media.” *SN Applied Sciences* 2(4):1–11. doi:10.1007/S42452-020-2492-Y/FIGURES/10.

Benabbouha, T., M. Siniti, H. El Attari, K. Chefira, F. Chibi, R. Nmila, and H. Rchid. 2018. “Red Algae Halopitys Incurvus Extract as a Green Corrosion Inhibitor of Carbon Steel in Hydrochloric Acid.” *Journal of Bio- and Tribo-Corrosion* 4(3):1–9. doi:10.1007/S40735-018-0161-0/METRICS.

Bouzenad, Nawal, Nesrine Ammouchi, Nadjla Chaib, Mohammed Messaoudi, Walid Bousabaa, Chawki Bensouici, Barbara Sawicka, Maria Atanassova, Sheikh F. Ahmad, and Wafa Zahnit. 2024. “Exploring Bioactive Components and Assessing Antioxidant and Antibacterial Activities in Five Seaweed Extracts from the Northeastern Coast of Algeria.” *Marine Drugs* 22(6):273. doi:10.3390/MD22060273/S1.

Brovko, Olga, Irina Palamarchuk, Natalia Gorshkova, Dmitriy Chukhchin, and Irina Eliseeva. 2025. “Effect of Polyelectrolyte Properties of Fucoidans on Antimicrobial Activity.” *Journal of Polymers and the Environment* 33(5):2138–48. doi:10.1007/S10924-025-03506-7/METRICS.

Cacciola, Nunzio Antonio, Paola De Cicco, Rebecca Amico, Fabrizia Sepe, Yan Li, Laura Grauso, Maria Francesca Nanì, Silvia Scarpato, Christian Zidorn, Alfonso Mangoni, and Francesca Borrelli. 2024. “Zosterabisphenone B, a New Diarylheptanoid Heterodimer from the Seagrass Zostera Marina, Induces Apoptosis Cell Death in Colon Cancer Cells and Reduces Tumour Growth in Mice.” *Phytotherapy Research* 38(8):4168–76. doi:10.1002/PTR.8269.

Dip, Md Reashad Raihan, Mohammad Khairul Alam Sobuj, Md Shoebul Islam, Alima Akter, Md Mehedi Hasan, Nazia Tasnim, Md Amdadul Haque, and S. M. Rafiquzzaman. 2024. “Phytochemicals, Antioxidant and Antibacterial Activity of Crude Extract of Sargassum Polycystum Collected from Bangladesh.” *Food and Humanity* 2:100278. doi:10.1016/J.FOOHUM.2024.100278.

Do, Manh Cuong, Tae Hyeon Yoon, Jeong Yong Moon, Gyung Min Go, and Somi Kim Cho. 2025. “Anticancer Effects of the Melosira Nummuloides Extract on Hepatocellular Carcinoma Cells through JAK2/STAT3 and MAPK Pathway Inhibition.” *Algal Research* 86:103949. doi:10.1016/J.ALGAL.2025.103949.

El-Din, Nihal G. Shams, Mohamed S. M. Abd El Hafez, Miral G. Abd El-Wahab, and Hassan A. H. Ibrahim. 2024. “Biological Activities of Derived Pigments and Polyphenols from the Newly Recorded Alga Phyllymenia Gibbesii.” *Scientific Reports 2024 14:1* 14(1):1–13. doi:10.1038/s41598-024-70825-5.

Farahin Ibrahim, Noradhiha, Aina Zulaikha Mohd Zuraimi, Wan Mohd Norsani Wan Nik, Wan Rafizah Wan Abdullah, and Mohammad Fakhratul Ridwan Zulkifli. 2024. “EVALUATION OF Caulerpa Lentilifera (SEAGRAPE) EXTRACT AS A POTENTIAL ANTI-CORROSION ADDITIVE IN EPOXY COATING FOR MILD STEEL PROTECTION IN THE MARINE ENVIRONMENT (Penilaian Ekstrak Caulerpa Lentilifera (Anggur Laut) Sebagai Bahan Tambahan Berpotensi Anti-Kakisan Dalam Salutan Epoksi Untuk Perlindungan Keluli Lembut Dalam Persekitaran Marin).” *Malaysian Journal of Analytical Sciences* 28:746–57.

Fernandes, Caio Machado, Thayssa Da, S. F. Fagundes, Nazir E. Dos Santos, Bruno Sergio Do Amaral, Quezia B. Cass, Alessandra L. Valverde, Júlio César, M. Silva, Odivaldo C. Alves, and Eduardo A. Ponzio. 2020. “Marine Octocoral Phyllogorgia Dilatata: Identification of Sesquiterpenes and Activity as a Natural and Renewable Corrosion Inhibitor.” *Anal. Bioanal. Electrochem* 12(4):437–57. www.abechem.com.

Fernández, Carolina, María Amparo Blanco Mendez, María Federica Faure, Ana Paula Murray, Silvana Rodríguez, Ailen Melisa Poza, Fernando Gaspar Dellatorre, and Leticia Andrea Fernández. 2025. “Macroalgal Extracts as an Alternative for the Control of Paenibacillus Larvae.” *Apidologie* 56(1):1–20. doi:10.1007/S13592-024-01127-Y/METRICS.

Frusciante, Luisa, Michela Geminiani, Alfonso Trezza, Tommaso Olmastroni, Pierfrancesco Mastroeni, Laura Salvini, Stefania Lamponi, Andrea Bernini, Daniela Grasso, Elena Dreassi, Ottavia Spiga, and Annalisa Santucci. 2024. “Phytochemical Composition, Anti-Inflammatory Property, and Anti-Atopic Effect of Chaetomorpha Linum Extract.” *Marine Drugs* 22(5):226. doi:10.3390/MD22050226/S1.

Fu, Xiaodan, Xinru Huang, Huizi Tan, Xiaojun Huang, and Shaoping Nie. 2024. “Regulatory Effect of Fucoidan Hydrolysates on Lipopolysaccharide-Induced Inflammation and Intestinal Barrier Dysfunction in Caco-2 and RAW264.7 Cells Co-Cultures.” *Foods* 13(22):3532. doi:10.3390/FOODS13223532/S1.

Gaber, Ghalia A., Mohamed Mohamady Ghobashy, Mohamed Madani, Dalal Mohamed Alshangiti, Sheikha A. Alkhursani, Samera Ali Al-Gahtany, and Norhan Nady. 2021. “Study of the Corrosion-Inhibiting Activity of the Green Materials of the Posidonia Oceanica Leaves’ Ethanolic Extract Based on PVP in Corrosive Media (1 M of HCl).” *Green Processing and Synthesis* 10(1):555–68. doi:10.1515/GPS-2021-0055/ASSET/GRAPHIC/J_GPS-2021-0055_FIG_009.JPG.

Gandhi, Grishma, Vijay Anand K. Gopalakrishnan, V. Veeragurunathan, and Arup Ghosh. 2024. “Unlocking the Potential of Tropical Red and Brown Seaweed-Based Biostimulants—a Comparative Assessment for Sustainable Maize (Zea Mays) Production.” *Journal of Applied Phycology* 36(3):1513–31. doi:10.1007/S10811-023-03155-0/METRICS.

Gangegoda, Sathya, Shamali Abeywardhana, Sachini Sigera, A. A. E. B. Nirmani, and Dinithi C. Peiris. 2024. “Antioxidant and Antimicrobial Properties of Codium Fragile (Suringar) Methanol Extract: Insights from Molecular Docking Analysis.” *Algal Research* 82:103619. doi:10.1016/J.ALGAL.2024.103619.

Gazali, Mohamad, Amir Husni, Anggris Prasiska Sukmadewi, Nurjanah, Muhammad Nursid, Yosie Andriani, Zuriat, Uswatun Hasanah, and Rina Syafitri. 2024. “Anticancer Activity of Marine Macroalgae Halimeda Tuna from Aceh Waters against Cervical Cancer Cells.” *Journal of Fisheries and Environment* 48(3):120–31. doi:10.34044/J.JFE.2024.48.3.10.

Gunathilaka, Thilina Lakmini, Hiruni S. Kumarasinghe, U. E. Bandaranayake, Maheshi Athapaththu, Kalpa W. Samarakoon, Pathmasiri Ranasinghe, and L. Dinithi C. Peiris. 2025. “Integration of In Vitro and In-Silico Analysis of Gracilaria Edulis on Anti-Cancer Potential and Apoptotic Signaling Pathway Activity.” *Cell Biochemistry and Biophysics* 1–19. doi:10.1007/S12013-025-01685-7/METRICS.

Hamad, Gamal M., Mukhtar M. F. Abushaala, Nisrin Besima Tarhuni, Taha Mehany, Raghda M. S. Moawad, Wahid I. El-Desoki, Mohamed A. H. Nagm El-diin, Mohammed A. Abd-Elmonem, Yasser El-Halmouch, Amany E. L. Sharkawy, Amr Amer, Sadeq K. Alhag, Laila A. Al-Shuraym, Ammar AL-Farga, and Asmaa H. M. Moneeb. 2025. “Yersinia Enterocolitica in Milk, Cheese, Yoghurt, and Ice Cream: Its Detection and Controlling by Some Marine Algal Extracts.” *LWT* 215:117184. doi:10.1016/J.LWT.2024.117184.

Hejna, Monika, Matteo Dell’Anno, Yanhong Liu, Luciana Rossi, Anna Aksmann, Grzegorz Pogorzelski, and Artur Jóźwik. 2024. “Assessment of the Antibacterial and Antioxidant Activities of Seaweed-Derived Extracts.” *Scientific Reports 2024 14:1* 14(1):1–15. doi:10.1038/s41598-024-71961-8.

Husni, Amir, Mohamad Gazali, Nurjanah Nurjanah, Rina Syafitri, Abdul Matin, and Zuriat Zuriat. 2024. “Cytotoxic Activity of Green Seaweed Halimeda Tuna Methanolic Extract Against Lung Cancer Cells.” *Journal of Multidisciplinary Applied Natural Science* 4(1):16–29. doi:10.47352/JMANS.2774-3047.172.

Je, Jun Geon, Chan Young Kim, Jaehak Sim, Yu Jin Roh, M. J. M. S. Kurera, N. M. Liyanage, Seungjin Jung, You Jin Jeon, and Bo Mi Ryu. 2024. “Anti-Inflammatory Activity and Structural Analysis of Fucoidan Extracted from Sargassum Fusiforme via ESI-TOF MS Spectrometry.” *Food Bioscience* 60:104414. doi:10.1016/J.FBIO.2024.104414.

Jeslina, V., S. J. Kirubavathy, A. Al-Hashem, S. Rajendran, C. Lacnjevac, and R. M. Joany. 2021. “Inhibitive Effect of an Alcoholic Extract of a Seaweed Sargassum Muticum in Controlling Corrosion of Mild Steel in 0.5 N HCl.” *Int. J. Corros. Scale Inhib* 10(4):1454–73. doi:10.17675/2305-6894-2021-10-4-6.

Jeslina, V., S. Jone Kirubavathy, Abdulhameed Al-Hashem, S. Rajendran, R. M. Joany, and Caslav Lacnjevac. 2023. “Mild Steel Corrosion Inhibition in 1 M HCl by an Alcoholic Extract of Sargassum Muticum.” *Portugaliae Electrochimica Acta* 41:151–65. doi:10.4152/pea.2023410204.

Jessima, S. J. Hepziba Magie, S. Subhashini, and James Arulraj. 2020. “Sunova Spirulina Powder as an Effective Environmentally Friendly Corrosion Inhibitor for Mild Steel in Acid Medium.” *Journal of Bio- and Tribo-Corrosion* 6(3):1–13. doi:10.1007/S40735-020-00370-X/METRICS.

Jo, Mi Jeong, Xiaotong Xu, Ji Eun Lee, Eui Baek Byun, Kwangwook Kim, Eui Hong Byun, and Dong Hyun Ahn. 2024. “Effect of Gamma-Irradiation on the Characteristics and Anti-Inflammatory Activity of Sargassum Fulvellum Water Extracts.” *Radiation Physics and Chemistry* 224:112072. doi:10.1016/J.RADPHYSCHEM.2024.112072.

Karaca, Sibel Altürk, and Elif Neyran Soylu. 2025. “Determination of the Antibacterial Activity of Microalgae Isolated from Giresun Streams.” *Aquatic Sciences and Engineering* 40(2):53–62. doi:10.26650/ASE20241596033.

Keshk, Ali A., Nadia H. Elsayed, Fahad M. Almutairi, Menier Al-Anazi, S. Said, Haitham M. Althurwi, Raghad K. Albalawi, and M. R. El-Aassar. 2024. “Effect of Green and Sustainable Extracted Fucoidan Polysaccharide as a Corrosion Inhibitor in 3.5% NaCl.” *Biomass Conversion and Biorefinery* 14(22):28219–32. doi:10.1007/S13399-022-03579-7/METRICS.

Kevrekidou, Alkistis, Andreana N. Assimopoulou, Varvara Trachana, Dimitrios Stagos, and Paraskevi Malea. 2024. “Antioxidant Activity, Inhibition of Intestinal Cancer Cell Growth and Polyphenolic Compounds of the Seagrass Posidonia Oceanica’s Extracts from Living Plants and Beach Casts.” *Marine Drugs* 22(3):130. doi:10.3390/MD22030130/S1.

Kherraz-Chemlal, D., F. Khelil, … M. Mazouzi-Polish Journal of, and undefined 2025. 2025. “Phytochemical Screening of Antioxidant and Antibacterial Activities of Marine Algae Extracts.” *Pjoes.ComD Kherraz-Chemlal, F Khelil, M Mazouzi, A Matallah-Boutiba, M BouderbalaPolish Journal of Environmental Studies, 2025•pjoes.Com*. doi:10.15244/pjoes/188450.

Korkmaz, Nuh. 2025. “Extract Optimization of Ulva Lactuca L. and Biological Activities of Optimized Extracts.” *BMC Biotechnology* 25(1):1–10. doi:10.1186/S12896-025-00954-W/TABLES/4.

Kraiem, Marwa, Sonia Ben Hamouda, Malek Eleroui, Marwa Ajala, Amal Feki, Amel Dghim, Zakaria Boujhoud, Marwa Bouhamed, Riadh Badraoui, Jean Marc Pujo, Khadija Essafi-Benkhadir, Hatem Kallel, and Ibtissem Ben Amara. 2024. “Anti-Inflammatory and Immunomodulatory Properties of a Crude Polysaccharide Derived from Green Seaweed Halimeda Tuna: Computational and Experimental Evidences.” *Marine Drugs 2024, Vol. 22, Page 85* 22(2):85. doi:10.3390/MD22020085.

Kumagai, Momochika, Akana Matsuda, Nozomi Shiiba, Tomoki Tsuruta, Hikaru Endo, Keisuke Nishikawa, and Yoshiki Morimoto. 2024. “Structure-Activity Relationship of Anti-Inflammatory Meroterpenoids Isolated from Dictyopteris Polypodioides in RAW264 Cells.” *Bioscience, Biotechnology, and Biochemistry* 88(6):594–600. doi:10.1093/BBB/ZBAE038.

Kumar, Selva, R. Selva Kumar, S. Gomathi, and V. Chandrasekaran. 2019. “Padina Pavonica Extract as a Green Inhibitor for Brass Corrosion in 0.1 N H3PO4 Solutions.” *Int. J. Adv. Sci. Eng* 6(2):1351. doi:10.29294/IJASE.6.2.2019.1351-1360.

Kumaran, T., Showket Yahya, G. Sudhandiran, P. Shanmugam, and K. Kathiravan. 2024. “Isolation, Structural Characterization, and Anti-Lung Cancer Evaluation of Fucoidan Isolated from the Brown Seaweed Turbinaria Decurrens.” *Applied Phycology* 5(1):1–12. doi:10.1080/26388081.2024.2365467.

Kurnia, Dewi Sri, and Noer Kasanah. 2025. “Bioprospecting of Indonesian Red Seaweeds Gracilaria Edulis and Pterocladiella Sp. 4: Anti-Vibrio Activity and Chemical Composition.” *Applied Phycology* 6(1):126–44. doi:10.1080/26388081.2025.2450829.

Lambert, Prescilla, Mahado Said-Ahmed, Charafeddine Jama, and Mounim Lebrini. 2023. “Molecules from Sargassum Algae as Green Inhibitor for C38 in HCl Medium: Extraction, Characterization and Electrochemical Study.” *Coatings* 13(12):2076. doi:10.3390/COATINGS13122076/S1.

Lau, Vincent, Fahrul Nurkolis, Moon Nyeo Park, Didik Setyo Heriyanto, Nurpudji Astuti Taslim, Trina Ekawati Tallei, Happy Kurnia Permatasari, Raymond R. Tjandrawinata, Seungjoon Moon, and Bonglee Kim. 2024. “Green Seaweed Caulerpa Racemosa as a Novel Non-Small Cell Lung Cancer Inhibitor in Overcoming Tyrosine Kinase Inhibitor Resistance: An Analysis Employing Network Pharmacology, Molecular Docking, and In Vitro Research.” *Marine Drugs* 22(6):272. doi:10.3390/MD22060272/S1.

Lee, Eun Gyeong, Sung Kun Yim, Sang Min Kang, Byung Jae Ahn, Chang Kwon Kim, Mina Lee, Dongseob Tark, and Gun Hee Lee. 2025. “Phlorofucofuroeckol-A: A Natural Compound with Potential to Attenuate Inflammatory Diseases Caused by Airborne Fine Dust.” *Medicina (Lithuania)* 61(1):165. doi:10.3390/MEDICINA61010165/S1.

Lee, Sang Hoon, Sang Seop Lee, Ga Young Lee, Seung Yun Han, Dong Sub Kim, Bong Ho Lee, and Yung Choon Yoo. 2025. “Endarachne Binghamiae Extract Ameliorates Inflammatory Responses in Macrophages Through Regulation of MAPK, NF-KB and PI3K/AKT Pathways, and Prevents Acute Lung Injury in Mice.” *Life 2025, Vol. 15, Page 88* 15(1):88. doi:10.3390/LIFE15010088.

Li, Zihao, Ziye Zhang, Yaqi Feng, Yatao Guo, Zhenxing Li, and Hong Lin. 2025. “Insight into the Anti-Inflammatory Sulfated Polysaccharides from Sargassum Carpophyllum and Structure-Function Relationships.” *Algal Research* 85:103841. doi:10.1016/J.ALGAL.2024.103841.

Lianlian, Ji, Xiu Weng, Zhishu Li, Guan-Yi Cao, and Minli Pan. 2025. “Tetraol, A New Bisabolane Sesquiterpene from Marine Alga-Sourced Aspergillus Taichungensis 299 and Its Antibacterial Activity against Common Pediatric Pathogens.” *Nat. Prod* 19:12. doi:10.25135/rnp.496.2501.3394.

Liu, Xin, Guanghong Luo, Ying Liu, Naibin Yang, and Wenqiao Yuan. 2025. “Antibacterial Actions of Algal Extract against Food Spoilage Bacteria Pseudomonas Fluorescens and Shewanella Putrefaciens.” *BioResources* 20(1):268. doi:10.15376/BIORES.20.1.268-281.

Lourenço-Lopes, C., M. Fraga-Corral, P. Garcia-Perez, A. Carreira-Casais, Aurora Silva, J. Simal-Gandara, and M. A. Prieto. 2022. “A HPLC‐DAD Method for Identifying and Estimating the Content of Fucoxanthin, Β‐carotene and Chlorophyll a in Brown Algal Extracts.” *Food Chemistry Advances* 1:100095. doi:10.1016/J.FOCHA.2022.100095.

M, Felemban Athary Abdulhaleem, Hussein S. Mohamed, Zuhir S. M. Akrim, Ahmed El-Morsy, Doaa E. Keshek, Khaled El‐Adl, and Shimaa K. Ali. 2025. “Antibacterial and Anti-Tumor Properties of Marine Posidonoia Oceanica Leaf Extracts.” *JHSMP* 31(3):268–90. doi:10.1080/10496475.2025.2478394.

Manmuan, Suwisit, Thanchanok Sirirak, Sukannika Tubtimsri, Arpa Petchsomrit, and Tiraniti Chuenbarn. 2025. “Phytochemical Analysis, Antioxidant Activity, and Cytotoxic Effects of Caulerpa Lentillifera Extracts Inducing Cell Apoptosis and Sub-G/G0-G1 Cell Cycle Arrest in KON Oral Cancer Cells.” *BMC Complementary Medicine and Therapies* 25(1):1–27. doi:10.1186/S12906-025-04835-9/FIGURES/14.

Martins, Margarida, Leonardo M. De Souza Mesquita, Bárbara M. C. Vaz, Ana C. R. V. Dias, Mario A. Torres-Acosta, Benoit Quéguineur, João A. P. Coutinho, and Sónia P. M. Ventura. 2021. “Extraction and Fractionation of Pigments from Saccharina Latissima (Linnaeus, 2006) Using an Ionic Liquid + Oil + Water System.” *ACS Sustainable Chemistry and Engineering* 9(19):6599–6612. doi:10.1021/ACSSUSCHEMENG.0C09110/ASSET/IMAGES/LARGE/SC0C09110_0010.JPEG.

McGurrin, Ailbhe, Rahel Suchintita Das, Arturo B. Soro, Julie Maguire, Noelia Flórez Fernández, Herminia Dominguez, Maria Dolores Torres, Brijesh K. Tiwari, and Marco Garcia-Vaquero. 2025. “Antimicrobial Activities of Polysaccharide-Rich Extracts from the Irish Seaweed Alaria Esculenta, Generated Using Green and Conventional Extraction Technologies, Against Foodborne Pathogens.” *Marine Drugs 2025, Vol. 23, Page 46* 23(1):46. doi:10.3390/MD23010046.

Melyon, Stacy, Pau Reig Rodrigo, Manon Sénard, Laura Brelle, Muriel Sylvestre, Sarra Gaspard, Drochss Pettry Valencia, and Gerardo Cebrian-Torrejon. 2024. “Investigating the Inhibitory Effect of Sargassum Natans and Sargassum Fluitans Extracts on Iron Corrosion in 1.00 Mol L−1 HCl Solution.” *Coatings* 14(10):1316. doi:10.3390/COATINGS14101316/S1.

Mendez, Rufa L., Stanislau Stanisheuski, Cristobal Miranda, Jan Frederik Stevens, and Jung Yeon Kwon. 2025. “Biofunctionalities of Seaweed Hydrolysates and Peptides from Pacific Dulse (Devaleraea Mollis): An in Vitro and in Silico Prospecting Approach.” *International Journal of Food Science and Technology* 60(1):11. doi:10.1093/IJFOOD/VVAE011.

Mohamad Yusof, Loqman, Hafandi Ahmad, Hasliza Abu Hassim, Farina Mustaffa-Kamal, Sharina Omar, Nur Karmila Zainundin, and Birdie Scott Padam. 2024. “A Study on the Impact of Diet Supplementation of Fermented Dried Seaweed Powder (Kappaphycus Alvarezii) on Healthy Cat Gut Performance, Skin and Hair Coat Conditions, and Behaviour.” *Veterinary Research Communications* 48(5):3061–72. doi:10.1007/S11259-024-10479-W/METRICS.

Mohamed, Shimaa I. A., Ghada H. Elsayed, Amgad El Shaffai, Shaymaa M. M. Yahya, and Walaa S. A. Mettwally. 2024. “In-Vitro Study of Cytotoxic and Apoptotic Potential of Thalassia Hemprichii (Ehren.) Asch. And Enhalus Acoroides (L.f.) Royle against Human Breast Cancer Cell Line (MCF-7) with Correlation to Their Chemical Profile.” *BMC Complementary Medicine and Therapies* 24(1):1–16. doi:10.1186/S12906-024-04512-3/FIGURES/9.

Mohamed, Shimaa I. A., Amgad El Shaffai, Shaymaa M. M. Yahya, Walaa S. A. Mettwally, and Ghada H. Elsayed. 2025. “Cytotoxicity and Apoptotic Effect of Methanolic Extract of Red Sea Seagrass, Enhalus Acoroides (Linn.f.) Royle, against Hepatocellular Carcinoma Cell Line (HepG-2) and Phytochemical Characterization.” *Advances in Traditional Medicine* 25(2):425–37. doi:10.1007/S13596-024-00775-W/METRICS.

Nadi, I., Z. Belattmania, B. Sabour, A. Reani, A. Sahibed-dine, C. Jama, and F. Bentiss. 2019. “Sargassum Muticum Extract Based on Alginate Biopolymer as a New Efficient Biological Corrosion Inhibitor for Carbon Steel in Hydrochloric Acid Pickling Environment: Gravimetric, Electrochemical and Surface Studies.” *International Journal of Biological Macromolecules* 141:137–49. doi:10.1016/J.IJBIOMAC.2019.08.253.

Navakanitworakul, Raphatphorn, Kesara Nittayaboon, Parinuch Chumkaew, Sinjai Phetcharat, and Jaruwan Mayakun. 2024. “Phytochemical Screening and Cytotoxic Activities of Enhalus Acoroides (L.f.) Royle and Halimeda Macroloba Decaisne on Cervical Cancer Cell Lines.” *Tropical Journal of Pharmaceutical Research* 23(12):2053–57. doi:10.4314/TJPR.V23I12.10.

Osman, Nehal A. H. K., Madeeha Arooj, Hee Kyoung Kang, Jin Won Hyun, and Young Sang Koh. 2024. “Chemical Composition, Antioxidant, and Anti-Inflammatory Properties of the Extracts of Hormophysa Triquetra and Padina Gymnospora.” *Natural Product Communications* 19(11). doi:10.1177/1934578X241298882/ASSET/4FDA66DB-30B8-4B09-9234-F8ED8EA28140/ASSETS/IMAGES/LARGE/10.1177_1934578X241298882-FIG5.JPG.

Osório, Catarina, Susana Machado, Juliana Peixoto, Sílvia Bessada, Filipa B. Pimentel, Rita C. Alves, and M. Beatriz P. P. Oliveira. 2020. “Pigments Content (Chlorophylls, Fucoxanthin and Phycobiliproteins) of Different Commercial Dried Algae.” *Separations 2020, Vol. 7, Page 33* 7(2):33. doi:10.3390/SEPARATIONS7020033.

Ouaddi, Oumaima, Abdallah Oukarroum, Rachid Bouharroud, Mohamed Alouani, Altaf EL blidi, Redouan Qessaoui, Nabil Radouane, Khaoula Errafii, Mohamed Hijri, Fatima Hamadi, and Mohammed Hassi. 2025. “A Study of Antibacterial Efficacy of Tetraselmis Rubens Extracts against Tomato Phytopathogenic Pseudomonas Corrugata.” *Algal Research* 86:103956. doi:10.1016/J.ALGAL.2025.103956.

Ozbil, Ertugrul, Mehmet Ilktac, Sultan Ogmen, Ovgu Isbilen, Jesus M. Duran Ramirez, Jana Gomez, Jennifer N. Walker, and Ender Volkan. 2024. “In Vitro Antibacterial, Antibiofilm Activities, and Phytochemical Properties of Posidonia Oceanica (L.) Delile: An Endemic Mediterranean Seagrass.” *Heliyon* 10(15):e35592. doi:10.1016/J.HELIYON.2024.E35592/ASSET/04088EB6-66BA-46CE-82EB-E3DD4C7E87DC/MAIN.ASSETS/MMCFIGS2.JPG.

Pan, Junzhi, Huayuan Liu, Xiaofeng Xu, Feijing Lv, Yu Qi, Chaojie Wang, Changle Wu, Zhongmin Sun, Gang Xu, and Pengcheng Yan. 2025. “Two New Chromane Meroditerpenoids with Anti-Neuroinflammatory Potential from a Chinese Collection of the Brown Alga Sargassum Siliquastrum.” *Natural Product Communications* 20(2). doi:10.1177/1934578X251321614/SUPPL_FILE/SJ-DOCX-1-NPX-10.1177_1934578X251321614.DOCX.

Paulo, Carolina, Joana Matos, Cláudia Afonso, and Carlos Cardoso. 2025. “Overcoming Extraction Hurdles and Assessing Biological Activity in a Major Invasive Seaweed Species in Europe, Rugulopteryx Okamurae.” *Marine Drugs 2025, Vol. 23, Page 141* 23(4):141. doi:10.3390/MD23040141.

Payet, Shamira, Bas de Vos, Maria Rose, Danilla Adonis, Aubrey Lesperance, and Thomas Hecht. 2024. “Unlocking the Therapeutic Treasures of Seagrasses: Antioxidant and Antimicrobial Activities of Halophila Stipulacea, Halodule Uninervis, and Thalassodendron Ciliatum.” *Western Indian Ocean Journal of Marine Science* 23(2):85–97. doi:10.4314/WIOJMS.V23I2.6.

Pereira, Tatiana, Sónia Barroso, Susana Mendes, Renata A. Amaral, Juliana R. Dias, Teresa Baptista, Jorge A. Saraiva, Nuno M. Alves, and Maria M. Gil. 2020. “Optimization of Phycobiliprotein Pigments Extraction from Red Algae Gracilaria Gracilis for Substitution of Synthetic Food Colorants.” *Food Chemistry* 321:126688. doi:10.1016/J.FOODCHEM.2020.126688.

Pereira, Tatiana, Sónia Barroso, Susana Mendes, and Maria M. Gil. 2020. “Stability, Kinetics, and Application Study of Phycobiliprotein Pigments Extracted from Red Algae Gracilaria Gracilis.” *Journal of Food Science* 85(10):3400–3405. doi:10.1111/1750-3841.15422.

Pérez-Álvarez, Sandra, Erick H. Ochoa-Chaparro, Julio César Anchondo-Páez, César M. Escobedo-Bonilla, Joel Rascón-Solano, Marco A. Magallanes-Tapia, Luisa Patricia Uranga-Valencia, Reinier Hernández-Campos, and Esteban Sánchez. 2024. “Nitrogen Assimilation, Biomass, and Yield in Response to Application of Algal Extracts, Rhizobium Sp., and Trichoderma Asperellum as Biofertilizers in Hybrid Maize.” *Nitrogen 2024, Vol. 5, Pages 1031-1047* 5(4):1031–47. doi:10.3390/NITROGEN5040066.

Perumal, Parthasarathi, Umamaheswari Arthanari, and Elumalai Sanniyasi. 2025. “Understanding the Molecular Mechanism of Emodin in Inhibiting Hepatocellular Carcinoma: An in Vitro and in Vivo Approach.” *Chemical Papers* 79(5):3381–95. doi:10.1007/S11696-025-04013-8/METRICS.

Prajoko, Yan Wisnu, Faqrizal Ria Qhabibi, Timothy Sahala Gerardo, Kanandya Kizzandy, Krisanto Tanjaya, Sebastian Emmanuel Willyanto, Happy Kurnia Permatasari, Reggie Surya, Nelly Mayulu, Nurpudji Astuti Taslim, Raymond Rubianto Tjandrawinata, Rony Abdi Syahputra, Trina Ekawati Tallei, Apollinaire Tsopmo, Bonglee Kim, Rudy Kurniawan, and Fahrul Nurkolis. 2024. “Revealing Novel Source of Breast Cancer Inhibitors from Seagrass Enhalus Acoroides: In Silico and In Vitro Studies.” *Molecules* 29(5):1082. doi:10.3390/MOLECULES29051082/S1.

Prisa, Domenico, and Marco Gobbino. 2021. “Microbic and Algae Biofertilizers in Aloe Barbadensis Miller.” *Open Access Research Journal of Biology and Pharmacy* 1(2):001–009. doi:10.53022/OARJBP.2021.1.2.0019.

Raj, Preethy P., Rajesh Kanna Gopal, and Elumalai Sanniyasi. 2024. “Investigating the Anti-Inflammatory and Anti-Arthritis Effects of Fucoidan from a Brown Seaweed.” *Current Research in Biotechnology* 7:100220. doi:10.1016/J.CRBIOT.2024.100220.

Rajasegaran, Rammiya, Asita Elengoe, Sau Pin Woo, Muhammad Ariffuddin Abd Hamd, Noorfatimah Yahaya, and Shahrul Hamid. 2024. “Analysis of Volatile Compounds in Caulerpa Lentillifera for Anti-Proliferative Studies in HEPG2 Liver Cancer Cells and in Silico Comparison.” *Pharmacognosy Research* 16(4):854–60. doi:10.5530/pres.16.4.97.

Ramezanpour, Zohreh, Fateme Ghanbari Pirbasti, and J. Robert Waaland. 2025. “Marine Algae Extract Effects on Cell Proliferation in a Malignant Melanoma Cell Line and an Immortalized Fibroblast Cell Line.” *Plant, Algae, and Environment* 9(1):62–77. doi:10.48308/PAE.2025.238286.1101.

Rhazzane, Nadia, Abdallah El-Asri, Moulay Driss Mellaoui, Aaziz Jmiai, Souad El Issami, Jaouad Abou Oualid, Karine Groenen‐Serrano, and Hanane Zejli. 2024. “Unveiling the Anti-Corrosion Potential of Seaweed Macroalga Codium Tomentosum: A Multifaceted Investigation into Its Mechanisms of Copper Protection.” *Journal of the Indian Chemical Society* 101(10):101266. doi:10.1016/J.JICS.2024.101266.

Rhazzane, Nadia, Aaziz Jmiai, Rachid El Brychy, and Hanane Zejli. 2023. “Corrosion Inhibition Potential of a Green Inhibitor ‘Red Algae’ for Copper in 1 M Sulfuric Acid Solution Determined by Electrochemical Measurement, Weight Loss Technique, UV–Visible, FTIR Spectroscopy and Complemented with Surface Analysis (SEM–EDS).” *Journal of Bio- and Tribo-Corrosion* 9(2):1–13. doi:10.1007/S40735-023-00747-8/METRICS.

Rifi, Mariam, Zeina Radwan, Nouha Sari-Chmayssem, Rayan Kassir, Ziad Fajloun, Abir Abdel Rahman, Marwan El-Sabban, Corinne Prévostel, Zeina Dassouki, and Hiba Mawlawi. 2025. “Exploring the Antineoplastic Properties of the Lebanese Jania Rubens Against Colorectal Cancer.” *Metabolites 2025, Vol. 15, Page 90* 15(2):90. doi:10.3390/METABO15020090.

Rossi, Raffaella, Francesco Vizzarri, Sabrina Ratti, Marisa Palazzo, Donato Casamassima, and Carlo Corino. 2020. “Effects of Long-Term Supplementation with Brown Seaweeds and Polyphenols in Rabbit on Meat Quality Parameters.” *Animals 2020, Vol. 10, Page 2443* 10(12):2443. doi:10.3390/ANI10122443.

Saluri, Mihkel, Margit Kaldmäe, and Rando Tuvikene. 2019. “Extraction and Quantification of Phycobiliproteins from the Red Alga Furcellaria Lumbricalis.” *Algal Research* 37:115–23. doi:10.1016/J.ALGAL.2018.11.013.

Samarasinghe, M. B., J. Sehested, M. R. Weisbjerg, M. Vestergaard, and L. E. Hernández-Castellano. 2021. “Milk Supplemented with Dried Seaweed Affects the Systemic Innate Immune Response in Preweaning Dairy Calves.” *Journal of Dairy Science* 104(3):3575–84. doi:10.3168/JDS.2020-19528.

Scaglia, Elena, Serena Reggi, Benedetta Canala, Sara Frazzini, Matteo Dell’Anno, Monika Hejna, and Luciana Rossi. 2023. “The Effects of Milk Replacer Supplemented with Ascophyllum Nodosum as a Novel Ingredient to Prevent Neonatal Diarrhea in Dairy Calves and Improve Their Health Status.” *Veterinary Sciences 2023, Vol. 10, Page 618* 10(10):618. doi:10.3390/VETSCI10100618.

Selim, Mohamed I., Tarek El-banna, Fatma Sonbol, Walaa A. Negm, and Engy Elekhnawy. 2025. “Unveiling the Potential of Spirulina Algal Extract as Promising Antibacterial and Antibiofilm Agent against Carbapenem-Resistant Klebsiella Pneumoniae: In Vitro and in Vivo Study.” *Microbial Cell Factories* 24(1):1–16. doi:10.1186/S12934-024-02619-3/FIGURES/12.

El Semary, Nermin, Amira Mohamed Abd El-Sattar, Eman Zakaria Ahmed, and Munirah Aldayel. 2023. “Mixotrophy of Algae: More Algal Biomass and More Biofertilization for Plants.” *Sustainability 2023, Vol. 15, Page 5815* 15(7):5815. doi:10.3390/SU15075815.

Sfriso, Andrea Augusto, Michele Gallo, and Franco Baldi. 2018. “Phycoerythrin Productivity and Diversity from Five Red Macroalgae.” *Journal of Applied Phycology* 30(4):2523–31. doi:10.1007/S10811-018-1440-3/METRICS.

de Sousa, Ana Carolina Sales Pereira, Jéssica Roberta Pereira Martins, Alanderson Arthu Araújo Alves, Sarah Sant’Anna Maranhão, Claudia Pessoa, Filipe Xavier Feitosa, Hosiberto Batista de Sant’Ana, and Ivanildo José da Silva. 2024. “Extraction and Characterization of R-Phycoerythrin from Wet and Lyophilized Macroalgae Solieria Filiformis by Pressurized Water Method.” *Algal Research* 80:103493. doi:10.1016/J.ALGAL.2024.103493.

Sparjan Samuvel, Rajan Marystella, Hashnu Dutta, Rajan Marystella Benisha, Kathirvel Muralidharan, Nishant Jain, Debasish Swain, and Vaikundamoorthy Ramalingam. 2024. “Chemopreventive Effects of Sargassum Cristaefolium against Prostate Cancer Targeting P53-Mediated Apoptosis Signaling Pathway: An in Vitro and in Vivo Study.” *Food Bioscience* 61:104839. doi:10.1016/J.FBIO.2024.104839.

Taba, Paulina, I. Sahidin, Nunuk Hariani Soekamto, and Corresponding Author. 2025. “Anticancer Assay of Methanol Extract of Gracilaria Salicornia Originating from the Hari Islands, Southeast Sulawesi, Against MCF-7 Cancer Cells.” *Journal of Community Health Provision* 5(1):14–25. doi:10.55885/JCHP.V5I1.553.

Thiraviyam, Pavithra, · Aafreen, · Kamala Kannan, Dhanraj Ganapathy, and · Pitchiah Sivaperumal. 2025. “Evaluation of Antioxidant and Antibacterial Activities of Seagrass Cymodocea Serrulata.” *Discover Plants 2025 2:1* 2(1):1–7. doi:10.1007/S44372-025-00147-X.

Tiarani, Yesha Ibanez, Oktora Susanti, Galuh Praka Siwi, Muhammad Nurwenta Kurniawan, Komang Putri Aryani, Nia Ramadhani, Moch Dhaffa, Abudhzar Ghyffary, Arsyah Poetra Lasmono, Tiara Safitri, Salsabila Tamaulina, Wanda Andella Putri, Marine Science, and Study Program. 2025. “Potential of Red Algae Eucheuma Cottoni as Antibacterial to Pseudomonas Aeruginosa.” *Jurnal Biologi Tropis* 25(1):440–47. doi:10.29303/JBT.V25I1.8272.

Trang Thuy, Nguyen Ngoc, and Tran Thanh Men. 2025. “Phytochemical and Bioactive Analysis of Extracted Brown Macroalgae (Dictyota Implexa) Collected in Vietnam.” *Biochemistry Research International* 2025(1):9461117. doi:10.1155/BRI/9461117.

Trigueros, Esther, Filipa Amaro, Paula Guedes de Pinho, and Andreia P. Oliveira. 2025. “Comprehensive Analysis of Dehydrated Edible Macroalgae: Volatile Compounds, Chemical Profiles, Biological Activities, and Cytotoxicity.” *Journal of Applied Phycology* 37(1):597–615. doi:10.1007/S10811-024-03403-X/FIGURES/5.

Trigueros, Esther, Andreia P. Oliveira, Paula B. Andrade, Romeu A. Videira, Paula Guedes de Pinho, M. Teresa Sanz, and Sagrario Beltrán. 2024. “Exploring the Bioactive Potential of Algae Residue Extract via Subcritical Water Extraction: Insights into Chemical Composition and Biological Activity.” *Food Chemistry* 458:140310. doi:10.1016/J.FOODCHEM.2024.140310.

Ulagesan, Selvakumari, Taek Jeong Nam, and Youn Hee Choi. 2021. “Extraction and Purification of R-Phycoerythrin Alpha Subunit from the Marine Red Algae Pyropia Yezoensis and Its Biological Activities.” *Molecules 2021, Vol. 26, Page 6479* 26(21):6479. doi:10.3390/MOLECULES26216479.

Verma, Nidhi, Krishnan D. Sehrawat, Poonam Mundlia, Anita R. Sehrawat, Ravish Choudhary, Vishnu D. Rajput, Tatiana Minkina, Eric D. van Hullebusch, Manzer H. Siddiqui, and Saud Alamri. 2021. “Potential Use of Ascophyllum Nodosum as a Biostimulant for Improving the Growth Performance of Vigna Aconitifolia (Jacq.) Marechal.” *Plants* 10(11):2361. doi:10.3390/PLANTS10112361/S1.

Vigors, Stafford, John O’doherty, Ruth Rattigan, and Torres Sweeney. 2021. “Effect of Supplementing Seaweed Extracts to Pigs until D35 Post-Weaning on Performance and Aspects of Intestinal Health.” *Marine Drugs* 19(4):183. doi:10.3390/MD19040183/S1.

Wang, Qihui, Xing Zhou, Xiaofeng Sun, Qi Zhang, Ruozhou Wang, Jinmei Zhao, Ruby Aslam, Yi Sun, Zhitao Yan, and Xueming Li. 2024. “Seaweed Extract as Green Corrosion Inhibitor for Carbon Steel in Hydrochloric Acid Solution.” *Colloids and Surfaces A: Physicochemical and Engineering Aspects* 700:134751. doi:10.1016/J.COLSURFA.2024.134751.

Wehbe, Nadine, Adnan Badran, Serine Baydoun, Ali Al-Sawalmih, Marc Maresca, Elias Baydoun, and Joelle Edward Mesmar. 2024. “The Antioxidant Potential and Anticancer Activity of Halodule Uninervis Ethanolic Extract against Triple-Negative Breast Cancer Cells.” *Antioxidants* 13(6):726. doi:10.3390/ANTIOX13060726/S1.

Widiastuti, Endang, and Mohammad Kanedi. 2025. “Anti-Plasmodial Activities of Ethanol Extract of Eucheuma Cottonii and Sargassum Polycystum Seaweeds Against Plasmodium Falciparum.” *Article in South Asian Research Journal of Biology and Applied Biosciences*. doi:10.36346/sarjbab.2025.v07i01.005.

Wu, Guan James, and Pei Wen Hsiao. 2024. “Assessment of Anti-Prostate Cancer Activity among Four Seaweeds, with Focus on Caulerpa Lentillifera J.Agardh.” *Foods 2024, Vol. 13, Page 1411* 13(9):1411. doi:10.3390/FOODS13091411.

Xavier, Bruno Toribio de Lima, Marcelo Franchin, Nima Mohammadi, Carolina Girotto Pressete, Lusânia Maria Greggi Antunes, Yandong Xu, Kai Wang, Jason Bennett, and Daniel Granato. 2024. “Designing Sustainable Antioxidant and Anti-Inflammatory Ingredients from Seaweeds for Functional Gummies.” *Future Foods* 10:100474. doi:10.1016/J.FUFO.2024.100474.

Yalçln, Sibel, Özge Karakaş, Emine Sükran Okudan, Kevser Sözgen Başkan, Sema Demirci Çekiç, and Reşat Apak. 2021. “HPLC Detection and Antioxidant Capacity Determination of Brown, Red and Green Algal Pigments in Seaweed Extracts.” *Journal of Chromatographic Science* 59(4):325–37. doi:10.1093/CHROMSCI/BMAA107.

Yohannan, Aron Santhosh Kumar, Palanisamy Mookkan, and Savithra Nagabhushana. 2025. “Phytochemistry, Bioactive Potential, and Chemical Characterization of Free-Floating Algae Ulva Profunda W.R.Taylor — a Lesser Known Species from Andhra Pradesh, India.” *Biomass Conversion and Biorefinery* 15(2):2223–41. doi:10.1007/S13399-023-05001-2/METRICS.

Yunianta, Yunianta, Rosalina Ariesta Laeliocattleya, Siti Narsito Wulan, and Yenny Risjani. 2025. “Fucoidan Extract from Brown Seaweed (Sargassum Echinocarpum): Molecular Weight, Elemental Composition, Selectivity and Anticancer Activity against Breast Cancer Cells.” *Natural Product Research* 39(11):3093–3101. doi:10.1080/14786419.2024.2326978.

Zheng, Dengdeng, and Guojie Wan. 2021. “Preparation of Algae Extract as Green Corrosion Inhibitor for Q235 Steel in Chloride Ion Solutions.” *International Journal of Electrochemical Science* 16(7):210734. doi:10.20964/2021.07.64.

Zin, May Thu, Thida Kaewkod, Jeeraporn Pekkoh, Wasu Pathom-aree, Supakit Chaipoot, Gochakorn Kanthakat, Phisit Seesuriyachan, Yan Yu Chen, Kuan Shiong Khoo, Benjamas Cheirsilp, and Sirasit Srinuanpan. 2025. “Integrated Deep Eutectic Solvent (DES) Extraction and Microwave-Assisted Process for Algal Protein Extraction: Process Improvement, Characterizations, Functional Properties, and Antioxidant and Anti-Colorectal Cancer Potentials.” *Journal of Agriculture and Food Research* 19:101673. doi:10.1016/J.JAFR.2025.101673.
